# Recent Advances in the Synthesis of Coumarin Derivatives from Different Starting Materials

**DOI:** 10.3390/biom10010151

**Published:** 2020-01-16

**Authors:** Melita Lončarić, Dajana Gašo-Sokač, Stela Jokić, Maja Molnar

**Affiliations:** Faculty of Food Faculty Osijek, Josip Juraj Strossmayer University, 31000 Osijek, Croatia; mloncaric@ptfos.hr (M.L.); dajana.gaso@ptfos.hr (D.G.-S.); sjokic@ptfos.hr (S.J.)

**Keywords:** synthesis, coumarins, Knoevenagel condensation, Pechmann reaction

## Abstract

The study of coumarin dates back to 1820 when coumarin was first extracted from tonka bean by Vogel. Compounds containing coumarin backbone are a very important group of compounds due to their usage in pharmacy and medicine. Properties and biological activities of coumarin derivatives have a significant role in the development of new drugs. Therefore, many different methods and techniques are developed in order to synthesize coumarin derivatives. Coumarin derivatives could be obtained from different starting materials with various methods but with big differences in yield. This review summarized various methods, techniques and reaction conditions for synthesis of coumarins from different compounds such as aldehydes, phenols, ketones and carboxylic acids.

## 1. Introduction

**Figure 1:**
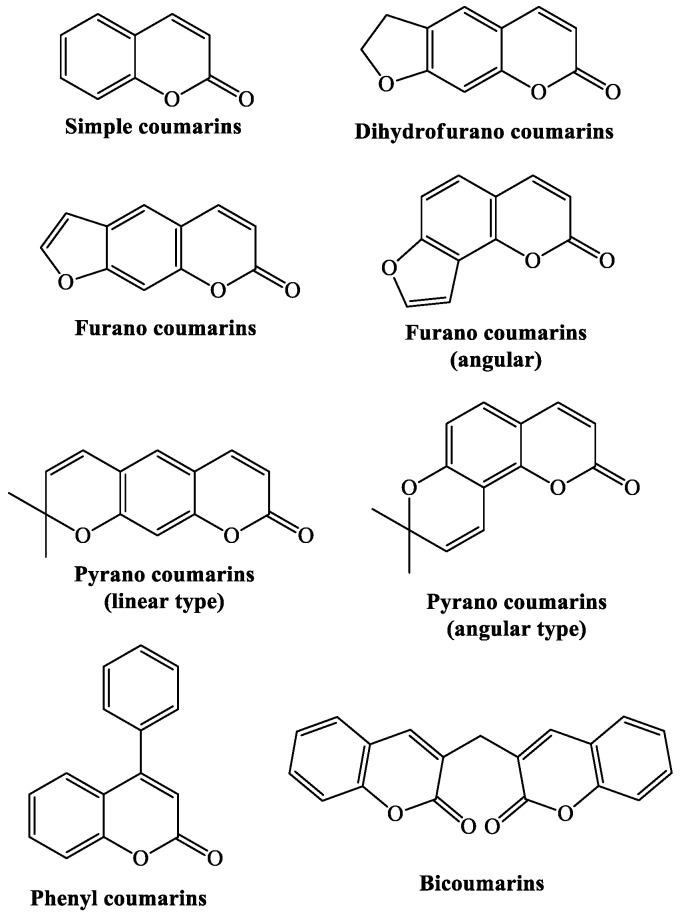
Six basic groups of natural coumarins.

Chemically, coumarins (2*H*-1-benzopyran-2-one) belong to the subgroup of lactones [1]. Coumarin is also known as 1,2-benzopyrone or *o*-hydroxycinnamic acid-8-lactone [2]. Natural coumarins can be divided into six basic groups as follows: simple coumarins, furanocoumarins, pyrano coumarins (linear type and angular type), dihydrofurano coumarins, phenyl coumarins and bicoumarins (Figure 1) [3]. First isolated parent coumarin was from tonka bean in (*Dipteryx odorata*) 1820 by Vogel [4,5]. The coumarins name originates from French word “Coumarou” for the tonka bean [6,7].

Coumarins are widely spread in the nature and can be found in many plant roots, flowers, leaves, peels, seeds and fruits, as secondary metabolites. Seo et al. and Bai et al. isolated more than 20 coumarins from the roots of *Angelica dahurica*, which has healing properties [8,9]. Iranshahi et al. reported isolation of three coumarins from roots *Ferula flabelliloba*, while Peng et al. isolated two new coumarins from the roots of *Clausena excavata* [10,11]. Six coumarins were extracted from *Ferulago suvelutina* and showed moderate antioxidant activity. Many coumarins are also distributed in different *Ferulago* species, mainly in the roots [12]. Coumarins are isolated from the plant flowers of various species such as *Bombax ceiba* [13], *Peltophorum pterocarpum* [14] and *Trifolium repens* [15]. Joselin et al. investigated the presence of phytochemical components in flowers of family *Apocynaceae.* Among four examined species (*Allamanda cathartica*, *Allamanda violacea*, *Wrightia tinctoria* and *Nerium oleander*), coumarins were detected in all flower extracts [16]. The plant leaves are also a good source of coumarin derivatives, which can be supported by numerous studies. Wang et al. extracted leaves of 11 bamboo species and determined 12 coumarin derivatives [17]. Coumarins with anti-inflammatory properties were isolated from the leaves of *Zanthoxylum schinifolium* [18] and *Zanthoxylum avicennae* [19]. Monoterpene coumarins, with major cytotoxic activity against *Leishmania major*, were isolated from *Micromelum minutum* leaves by Sakunpak et al. [20]. Coumarins are isolated from leaves of following species: *Matricaria chamomilla* L. [21], *Murraya paniculata* [22], *Bambusa pervariabilis* [23] and *Calophyllum inophyllum* [24]. Seeds are also a good source of different coumarins. Razavi and coworkers isolated furanocoumarin imperatorin and two other coumarins from *Zosima absinthifolia* seeds [25]. Several sesquiterpene coumarins were extracted from the seeds of *Ferula sinkiangensis* [26,27]. From the extract of the *Cnidium monnieri* L. seeds, coumarins osthole, xanthotoxin and imperatorin were isolated [28]. Regarding the coumarins found in plant peel, most of them are extracted from citrus peels [29,30,31].

The above mentioned plants are just a small part of the plant species that are rich in coumarins. Most of the extracted coumarins have biological activity and therefore coumarin derivatives are increasingly being synthesized, since their extraction from plants is time-consuming and unprofitable (too many operation steps to the final product) [32]. Coumarins could be synthesized with many different methods like Perkin reaction, Knoevenagel condensation, Pechmann condensation, Wittig reaction, Baylis-Hillman reaction, Claisen rearrangement and Vilsmeier-Haack and Suzuki cross-coupling reaction [33,34]. Many authors have published researches exploring the medicinal properties of coumarins [4,6,12,35,36,37]. Coumarins possess antimicrobial activity such as antibacterial [38,39,40,41,42,43,44] and antifungal [40,45,46,47,48]. Numerous coumarin derivatives showed significant antioxidant activity [49,50,51,52,53]. Some coumarins are derived as acetylcholinesterase (AchE) inhibitors, which could be considered as a drug in Alzheimer disease treatment [54,55,56]. There are many other biological activities that are associated with coumarins such as: anti-inflammatory [57,58], anti-HIV [6,59], anticancer [8,53,60], antituberculosis [61], anticoagulant [62], antiviral [63] and antihyperglycemic [64].

As coumarins have proven to be effective pharmacophore, there is a growing demand for their synthesis. Many methods have been employed for their synthesis, but each method includes various starting materials and different reaction conditions. Present article summarizes the recently reported researches, where different components, techniques and methods were used in the coumarin derivatives synthesis.

## 2. Coumarin Derivatives Synthesized from Aldehydes

**Scheme 1:**
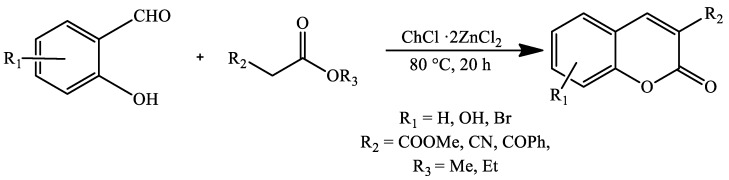
Green synthesis of coumarin derivatives in deep eutectic solvent.

Keshavarzipour and Tavakol [65] disclosed the green synthesis of coumarin derivatives in deep eutectic solvent (DES) by Knoevenagel condensation. DES was prepared by mixing choline chloride (ChCl) and zinc chloride at 100 °C. For the synthesis of coumarins derivatives, simple or substituted salicylaldehydes (4-hydroxy, 5-bromo) and methylene compounds (dimethyl malonate, ethyl cyanoacetate, ethyl 3-oxo-3-phenylpropanoate) were used (Scheme 1). The yields were high (61–96%) and DES acted both as a solvent and as catalyst.

**Scheme 2:**
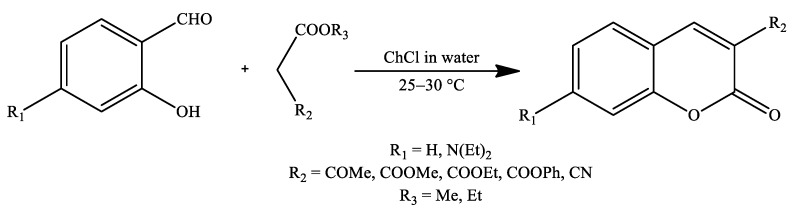
Coumarin derivatives obtained in aqueous media with ChCl as catalyst.

Phadtare and Shankarling carried out the reactions in order to obtain coumarin derivatives [66]. All mixtures were stirred at 25–30 °C in aqueous media. Syntheses were performed by Knoevenagel condensation from substituted aldehydes and active methylene compounds in the presence of ChCl as a catalyst (Scheme 2). Yields of the synthesized coumarin derivatives were in the range from 79 to 98%.

**Scheme 3:**
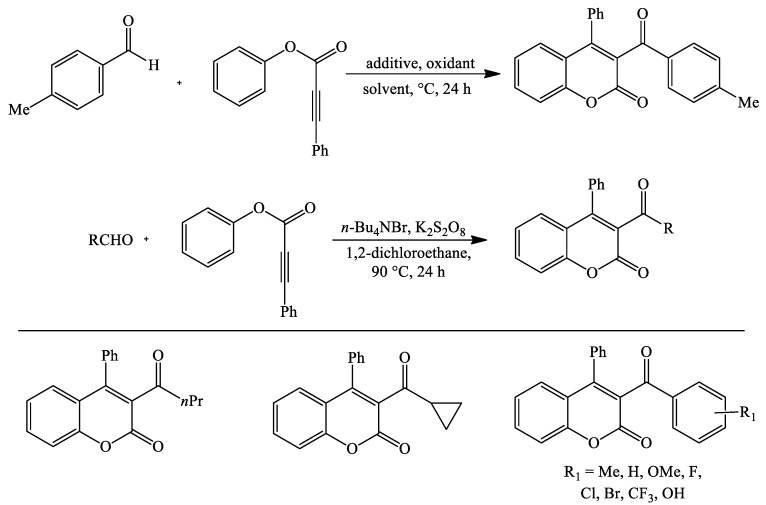
Cyclization reaction of phenyl 3-phenylpropiolate with various aldehydes.

**Scheme 4:**
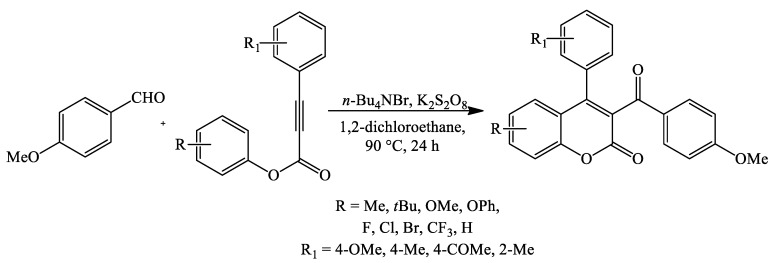
Cyclization reactions of 4-methoxybenzaldehyde and various alkynoates.

Mi and co-workers described metal-free tandem oxidative acylation and cyclization between alkynoates and aldehydes in 3-acyl-4-aryl preparation [67]. Reaction between phenyl 3-phenylpropionate and diethyl *p*-tolualdehyde was conducted in order to obtain optimized reaction conditions. During reaction optimization, following parameters has been changed: additive (pivalic acid, *n*-Bu_4_NF, *n*-Bu_4_NCl, *n*-Bu_4_NBr, *n*-Bu_4_NI and Et_4_NBr), oxidant (*tert*-butyl hydroperoxide, K_2_S_2_O_8_, Na_2_S_2_O_8_ and (NH_4_)_2_S_2_O_8_), solvent (1,2-dichloroethane, MeCN, dioxane, toluene, chlorobenzene and H_2_O) and temperature (80, 90 and 100 °C). All reactions were carried out in the sealed tubes under N_2_ in oil bath for 24 h. K_2_S_2_O_8_ as oxidant_,_ *n*-Bu_4_NBr as additive, 1,2-dichlorethane as solvent and temperature of 90 °C were found to be the best reaction conditions. Cyclization reaction of phenyl 3-phenylpropiolate with various aldehydes was carried out in optimized conditions (Scheme 3). In Scheme 4 cyclization reactions of 4-methoxybenzaldehyde and various alkynoates are shown.

**Scheme 5:**
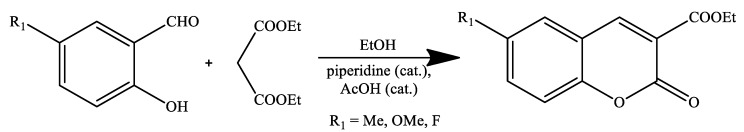
Synthesis of coumarin derivatives in the presence of piperidine and acetic acid as catalysts.

Suljić and Pietruszka have reported the synthesis of coumarins by Knoevenagel condensation of salicylaldehyde and diethyl malonate in EtOH (Scheme 5) [68]. Piperidine and acetic acid were added as catalysts. From the synthesized coumarins, chromanones were obtained in the process of continuous flow hydrogenation. Further, chromanones were reacted with a number of catehols in laccase-catalyzed arylation.

**Scheme 6:**
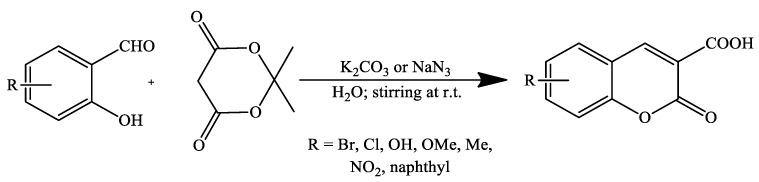
One-pot synthesis of coumarin-3-carboxylic acids in water.

Brahmachari reported one-pot synthesis of coumarin-3-carboxylic acids via Knoevenagel condensation [69]. Syntheses were performed at room temperature (RT) in water. First, model reaction between salicylaldehyde and Meldrum’s acid was conducted in order to find the catalyst to obtain high yields of the product. The best catalysts were proven to be sodium azide and potassium carbonate giving the product yield of 99 and 92%, respectively. Various substituted salicylaldehydes and Meldrum’s acid were put in reaction with both catalysts and afforded number of substituted coumarin-3-carboxylic acids (Scheme 6) in yields 73–99%. It should be noted that sodium azide is acutely very toxic, especially in the amounts in which it is added in the reactions (50 mol%). Therefore, a method with potassium carbonate (20 mol%) as a catalyst is recommended. 

**Scheme 7:**
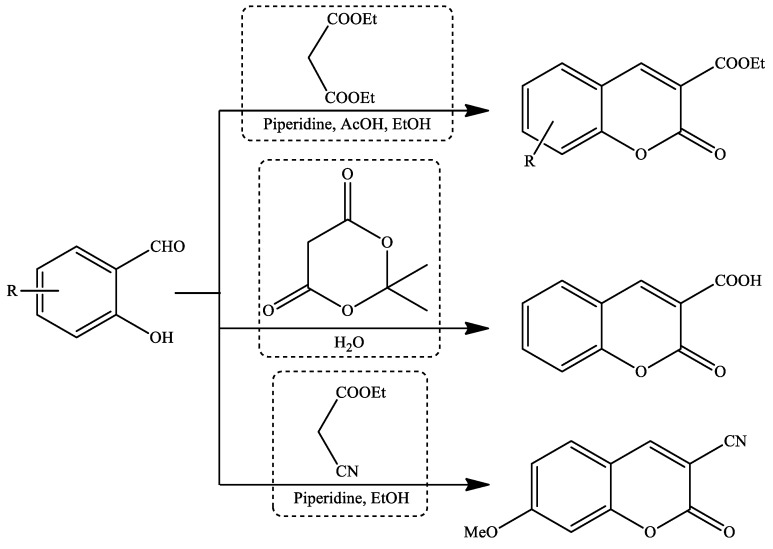
Synthesis of different coumarins from active methylene compounds and salicylaldehydes.

Silveira Pinto and Souza performed a synthesis of different coumarins from active methylene compounds (diethylmalonate, Meldrum’s acid and ethylcyanoacetate) and salicylaldehydes by Knoevenagel condensation (Scheme 7) [70]. The reaction was carried out in absolute EtOH or H_2_O with addition of piperidine and glacial AcOH. Model reaction of salicylaldehyde and diethyl malonate using heating and ultrasonic method was performed. Comparison of the ultrasonic and reflux procedure shows higher yield and shorter reaction time in case of ultrasound irradiation (40 min compared with 7 h). Using ultrasound irradiation at a frequency of 20 kHz with 90% power output and without pulsing, variety coumarins were obtained.

**Scheme 8:**
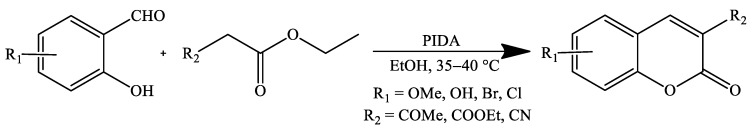
Phenyliododiacetate mediated reaction of salicylaldehydes and α-substituted ethylacetates.

Khan et al. reported an efficient synthesis of coumarin derivatives via Knoevenagel condensation [71]. Reaction parameters (solvent, catalyst) were changed in order to find the best reaction conditions in the terms of yield. After optimization, reaction conditions that turned out to be the best were: phenyliododiacetate (PIDA) mediated reaction in EtOH at 35–40 °C, which afforded yield of 90–92%. Under optimum conditions reactions of salicylaldehydes and *α*-substituted ethylacetates (Scheme 8) afforded product yields of 80–92%.

**Scheme 9:**
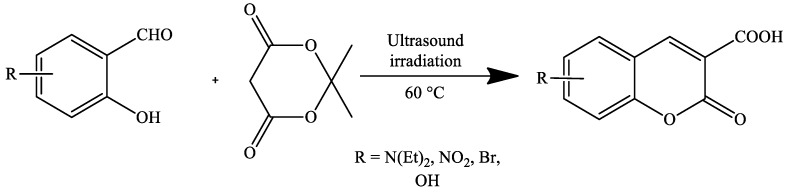
Green method for synthesis of coumarin-3-carboxylic acids under ultrasound irradiation.

Fiorito et al. developed a green method for the synthesis of coumarin-3-carboxylic acids by Knoevenagel condensation [72]. This new method involved the use of solvents such vegetable juices, liqueur limoncello and waste waters deriving from processing of olive and buttermilk. Reactions of substituted salicylaldehydes and Meldrum’s acid were carried out under the ultrasound irradiation at 60 °C (Scheme 9). Products were obtained in very good yields (91–99%) and the best conversions were noticed in lemon juice.

**Scheme 10:**
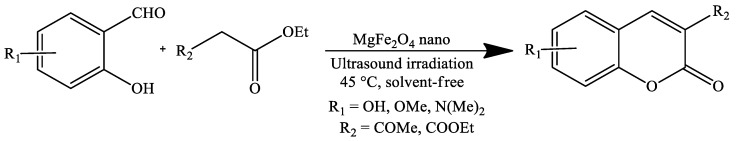
Synthesis of 3-substituted coumarins between salicylaldehydes and 1,3-dicarbonyl compounds in presence of nano MgFe_2_O_4_.

Solvent-free Knoevenagel condensation was performed in order to synthesize 3-substituted coumarins by Ghomi and Akbarzadeh [73]. In order to optimize reaction conditions, different solvents (DMF, MeOH, MeCN, EtOH and no solvent) and catalysts (nano CuO, nano MgO, nano ZnO and nano MgFe_2_O_4_) were used. Green synthesis in presence of MgFe_2_O_4_ nanoparticles was conducted between various salicylaldehydes and 1,3-dicarbonyl compounds at 45 °C by ultrasound irradiation (Scheme 10). 3-Substituted coumarins were obtained in good yields 63–73%. 

**Scheme 11:**
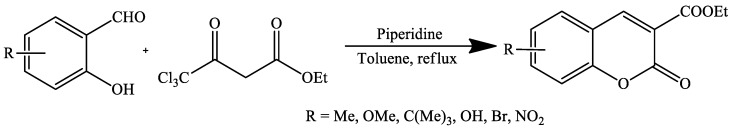
Synthesis of 2H-chromene-3-carboxylates in presence of piperidine in toluene.

2*H*-Chromene-3-carboxylates were synthesized in the reaction of salicylaldehydes and 4,4,4-trichloro-3-oxobutanoate via Knoevenagel pathway by Sairam et al. [74]. Optimization of reaction conditions was conducted employing various solvents (EtOH, DCM, DMF, MeCN, THF, ether, MeOH and toluene) and catalysts (piperidine, PPh_3_, DABCO, DMAP, N(C_2_H_5_)_3_, pyridine, imidazole, EtN(Pr)_2_, 2,6-lutidine, NaOMe, KOH and K_2_CO_3_). A series of coumarin derivatives was synthesized under optimal conditions (toluene and piperidine) as shown in Scheme 11. Yields of the obtained coumarins were in the range from 25 to 82%.

**Scheme 12:**
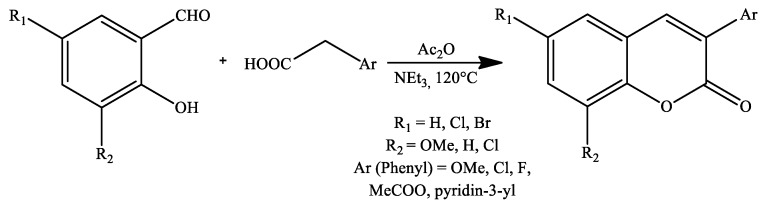
Synthesis of 3-arylcoumarins via Perkin condensation in the presence of anhydride and trimethylamine.

3-Arylcoumarins were prepared via Perkin condensation from salicylaldehydes and phenylacetic acids as starting materials (Scheme 12). Reactions were carried out in the presence of acetic anhydride and triethylamine at 120 °C with stirring. After purification, 3-arylcoumarins were obtained in moderate to good yields (46–74%) [58].

**Scheme 13:**
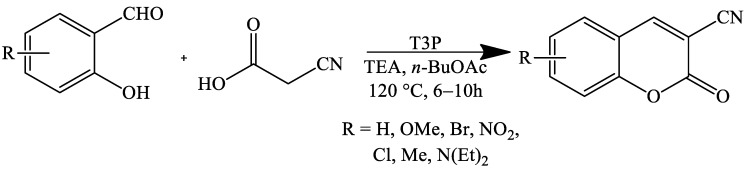
One-pot synthesis of cyanocoumarins in presence of T3P, trimethylamine and butyl acetate.

**Scheme 14:**
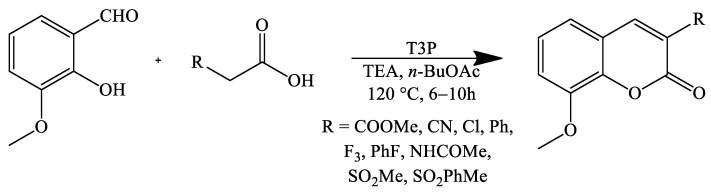
Synthesis of coumarin derivatives trough reaction of carboxylic acids and 2-hydroxy-3-methoxybenzaldehyde.

Augustine and co-workers developed one-pot synthesis of substituted coumarins via Perkin condensation [75]. Reaction of salicylaldehyde and cyanoacetic acid was chosen as a model reaction. Eight reactions with different parameters (time, temperature, molarity of propylphosphonic anhydride T3P) were performed in order to obtain the best reaction conditions. All reactions were conducted in the presence of T3P, TEA (triethylamine) and n-BuOAc (butyl acetate). Further, various substituted salicylaldehydes were reacted with cyanoacetic acid to afford coumarin derivatives (Scheme 13). In addition, coumarin derivatives were synthesized trough reaction of various carboxylic acids and 2-hydroxy-3-methoxybenzaldehyde under optimized conditions (Scheme 14).

**Scheme 15:**
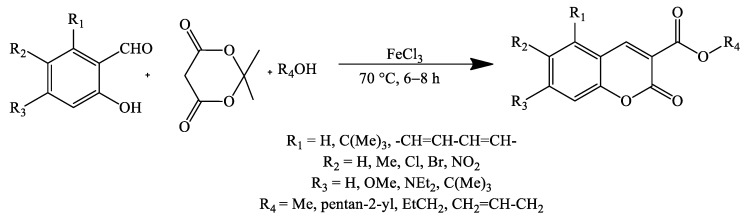
Synthesis of coumarin-3-carboxylic esters in multicomponent reaction in the presence of FeCl_3_.

In order to synthesize coumrin-3-carboxylic esters multicomponent reactions was performed by He et al. [76]. Screening of the catalysts was performed (Cu(OAc)_2_, CuBr, CuSO_4_, NiCl_2_·6H_2_O, AgNO_3_, K3Fe(CN)_6_ and FeCl_3_) and FeCl_3_ was proven to be the best. Optimization of the reaction conditions included different solvents (EtOH, DMF, THF, MeCN, toluene, DMSO, cyclohexane and H_2_O) and temperatures (RT, 50, 70 and 100 °C). The highest yield of 93% was obtained in EtOH at 70 °C. These parameters were applied to perform the reaction of substituted salicylaldehydes, Meldrum’s acid and various alcohols (Scheme 15). Coumarin-3-carboxylic acids were obtained in good to excellent yields (73–91%).

**Scheme 16:**
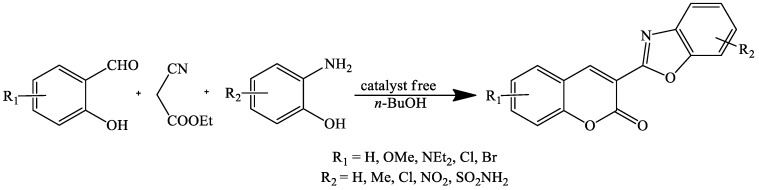
Three-component one-pot synthesis of 3-benzoxazole coumarins.

Three-component one-pot synthesis of coumarin derivatives was developed by Jiang et al. [77]. Trial reaction of salicylaldehyde, ethyl cyanoacetate and *o*-aminophenol was performed in order to find a suitable solvent (MeOH, EtOH, BuOH and amyl alcohol). The highest yield of 66% was afforded in *n*-BuOH. Substituted salicylaldehydes, ethyl cyanoacetate and *o*-aminophenols (Scheme 16) were reacted to afford 3-benzoxazole coumarins, which are obtained in yields 40–79%. 

**Scheme 17:**
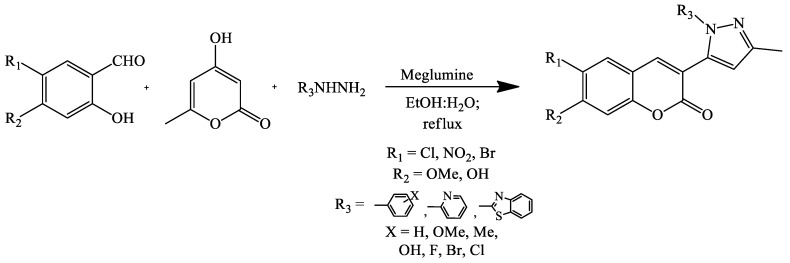
Synthesis of pyrazolylcoumarins in a multicomponent reaction.

Pyrazolylcoumarins were synthesized by Li et al., [78] under conditions that are established to be the best in previously reaction optimization. Different solvents (EtOH, EtOH:H_2_O (1:1), H_2_O, glycerin, MeCN and deep eutectic solvents), catalysts (without catalyst, Fe_2_O_3_, L-proline, CaCO_3_, piperidine, 1,3-dimethylurea, chitosan, betaine HCl, mannitol, DABCO and meglumine) and temperatures (reflux, 80 °C) were applied in order to find the best conditions for pyrazolylcoumarins synthesis in term of the highest yield. The highest yield of 80% was obtained under following conditions: EtOH:H_2_O (1:1), meglumine, reflux. A series of pyrazolylcoumarins were synthesized in one-pot three-component reaction between various salicylaldehydes, 4-hydroxy-6-methyl-2*H*-pyran 2-one and hydrazines (Scheme 17). Products were synthesized in good to excellent yields (70–89%).

**Scheme 18:**
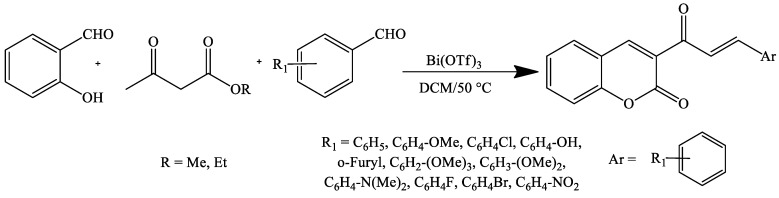
Synthesis of coumarin-chalcone compounds in multicomponent reaction.

One-pot three-component reactions were performed between salicylaldehyde, α-ketoester and various aromatic aldehydes (Scheme 18) [79]. Several reactions were performed in order to optimize reaction conditions changing catalyst (FeCl_3_·6H_2_O, Fe(OTf)_3_, ZnBr_2_, Zn(OTf)_2_, MgCl_2_, Mg(OTf)_2_, CuCl_2_, Cu(OTf)_2_ and Bi(OTf)_3_) and solvent (MeOH, *N*, *N*-dimethylformamide, toluene, MeCN and DCM). The highest yield of 96% was obtained in presence of Bi(OTf)_3_ in DCM at 50 °C. Coumarin-chalcone compounds were obtained in excellent yields (88–96%). 

**Scheme 19:**
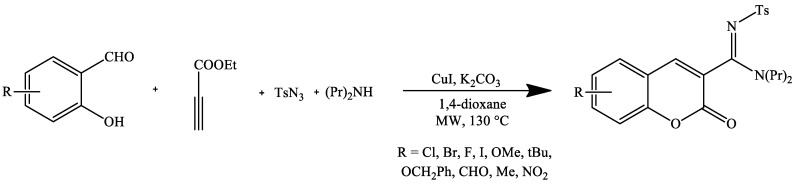
Microwave assisted four-component synthesis of 3-N-sulfonylamidine coumarins.

Murugavel and Punniyamurthy reported microwave assisted four-component synthesis of 3-*N*-sulfonylamidine coumarins [80]. Model reaction was conducted between salicylaldehyde, ethyl propiolate, tosyl azide and diisopropylamine in the presence of different copper salts (CuI, CuBr, CuCl and Cu(acac)_2_), bases (K_3_PO_4_, K_2_CO_3_, Cs_2_CO_3_, Na_2_CO_3_ and DBU) and solvents (1,4-dioxane, DMSO, toluene and DMF). The best reaction conditions were proven to be in the presence of CuI, K_2_CO_3_ and dioxane, series of coumarins was synthesized with moderate to high yields (Scheme 19).

**Scheme 20:**
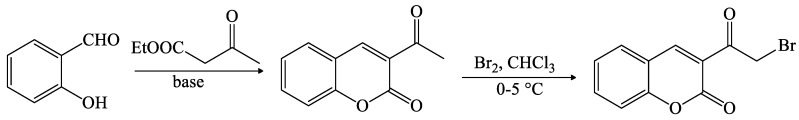
Two-step synthesis of bromoacetylcoumarin under microwave irradiation.

Osman et al. reported a two-step synthesis of bromoacetylcoumarin by microwave irradiation [81]. The model reaction was carried out in EtOH or solvent-free at 50 °C or 120 °C. Piperidine and L-proline were used as a base. Yield of 99% was obtained in EtOH with piperidine at 50 °C. First step was the synthesis of 3-acetylcoumarin from salicylaldehyde and ethylacetoacetate. Second reaction was electrophilic bromination of 3-acetylcoumarin in CHCl_3_ (Scheme 20).

**Scheme 21:**
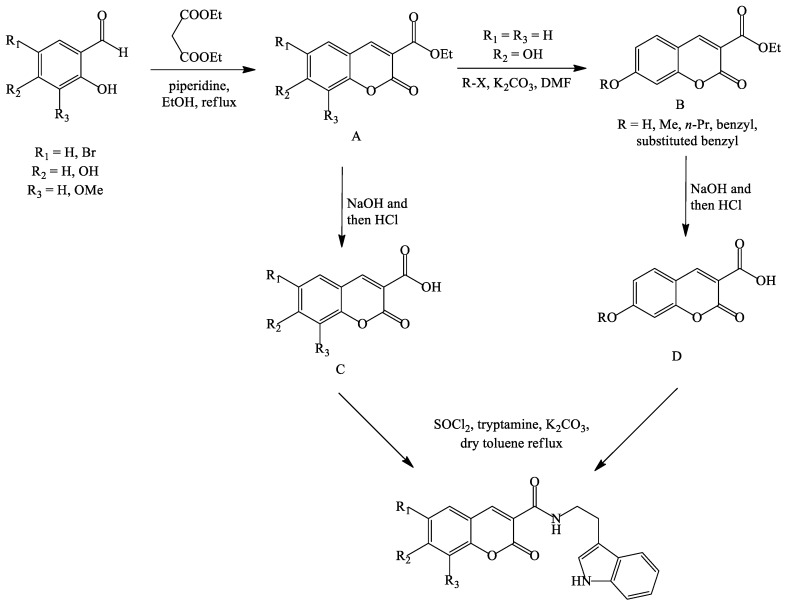
Three and four-step synthesis of N-(2-(1H-indol-3-yl)ethyl)-2-oxo-2H-chromene-3-carboxamides.

Ghanei-Nasab et al. developed three and four-step synthesis of *N*-(2-(1*H*-indol-3-yl)ethyl)-2-oxo-2*H*-chromene-3-carboxamides, which were tested against AChE and BuChE (Scheme 21) [82]. In the first step salicylaldehyde derivatives, diethyl malonate and piperidine were refluxed in EtOH and ethyl coumarins-3-carboxylates were obtained (compounds **A**). 7-Hydroxy derivative was reacted with alkyl halides or benzyl derivatives by using potassium carbonate in DMF to obtain *O*-alkyl or *O*-benzyl derivatives (compounds **B**). All obtained ethyl esters were hydrolyzed in the presence of sodium hydroxide to give coumarins-3-carboxylic acids (compounds **C** and **D**). Coumarins-3-carboxylic acids were converted to acid chlorides using thionyl chloride. Final products were obtained in reaction of acid chlorides and tryptamine in the presence of potassium carbonate in dry toluene. In order to decrease the reaction time, the last step of the reaction was carried out under different conditions (microwave irradiation in different solvents; solvent-free conditions). The best results were achieved by using MeCN as a solvent under microwave irradiation, where reaction time was decreased from 15 h to 5 min in comparison to conventional conditions.

**Scheme 22:**
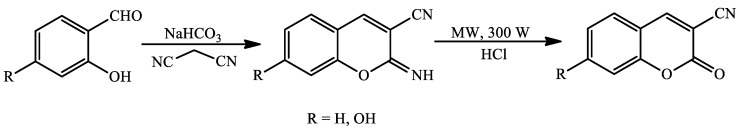
Two-step synthesis of substituted 3-cyanocoumarins under microwave irradiation.

Rabahi et al. synthesized coumarins in a two-step reaction from salicylaldehydes and malononitrile [83]. In the first step, iminocoumarins were obtained from salicylaldehyde and malonitrile in the presence of NaHCO_3_. Further, iminocoumarins were hydrolyzed in the presence of HCl under microwave irradiation. Both reaction steps are shown in Scheme 22. 

**Scheme 23:**
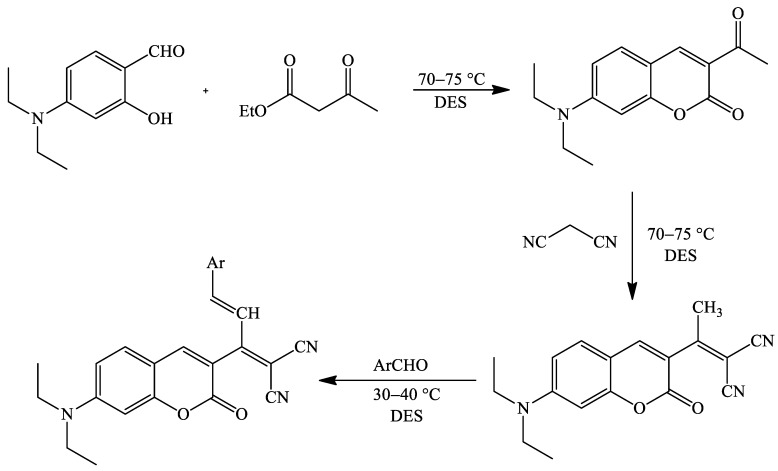
One-pot synthesis of fluorescent colorant in deep eutectic solvents.

Phadtare et al. developed synthesis of fluorescent colorant based on cyanocoumarins. One-pot synthesis was applied using deep eutectic solvent (DES) (ChCl:urea) [84]. In the first step 4-(diethylamino)-2-hydroxybenzaldehyde and ethyl acetoacetate were mixed and stirred in DES. After reaction completion, 3-acetyl-7-(diethylamino)-2*H*-chromen-2-one was obtained and malononitrile was added. The last step included addition of different aromatic aldehydes in order to obtain fluorescent colorant. All reaction steps are shown in Scheme 23.

**Scheme 24:**
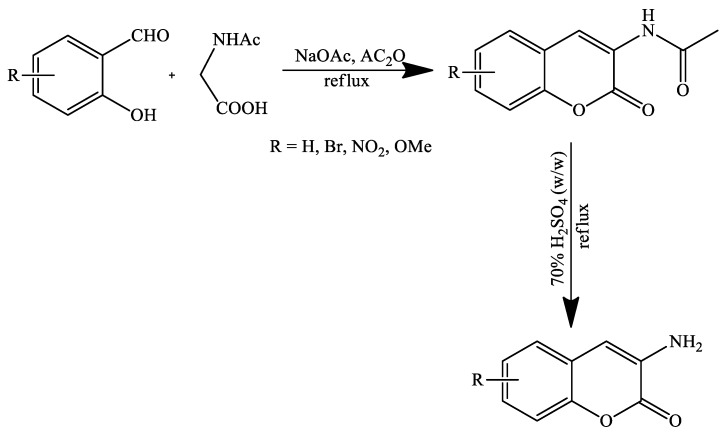
Synthesis of substituted 3-aminocoumarins in two-step reaction.

**Scheme 25:**
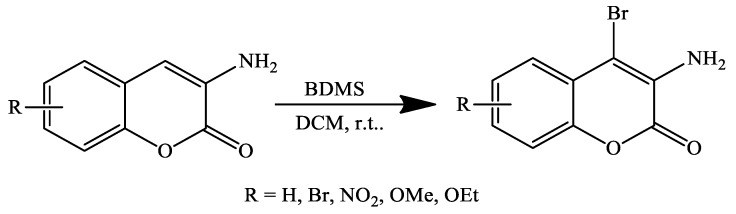
Synthesis of substituted 3-amino-4-bromocoumarins in the presence of bromodimethylsulfonium bromide.

Das et al. have developed procedure for synthesis of 3-amino-4-bromocoumarin [85]. Initially, reactions of various salicylaldehydes and *N*-acetylglycine were conducted in order to obtain 3-acetamidocoumarins, followed by hydrolysis to afford 3-aminocoumarins (Scheme 24). As brominating reagent bromodimethylsulfonium bromide (BDMS) was used. Reactions of 3-aminocoumarins and BDMS were performed in DCM at room temperature (Scheme 25). Yields of brominated compounds were 84–92%.

**Scheme 26:**
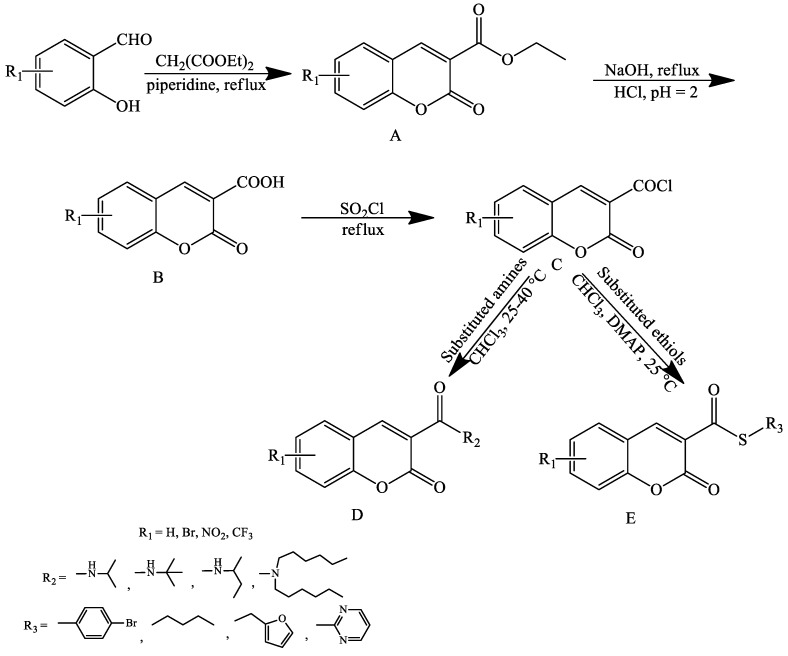
Four-step reaction in synthesis of coumarin derivatives.

He et al. reported synthesis of different coumarin derivatives starting with the condensation of the substituted salicylaldehydes and malonate in the presence of piperidine [86]. In order to synthesize component **B**, obtained coumarins **A** were treated with sodium hydroxide and hydrochloric acid. Reactions of components **B** and thionyl chloride afforded compounds **C**. 2-Oxo-2*H*-chromene-3-carbonyl chlorides (**C**) were reacted with 4-dimethylaminopyridine or substituted ethiols in order to obtain compounds **D** or **E**, respectively. All synthetic steps and reaction conditions are shown in the Scheme 26.

**Scheme 27:**
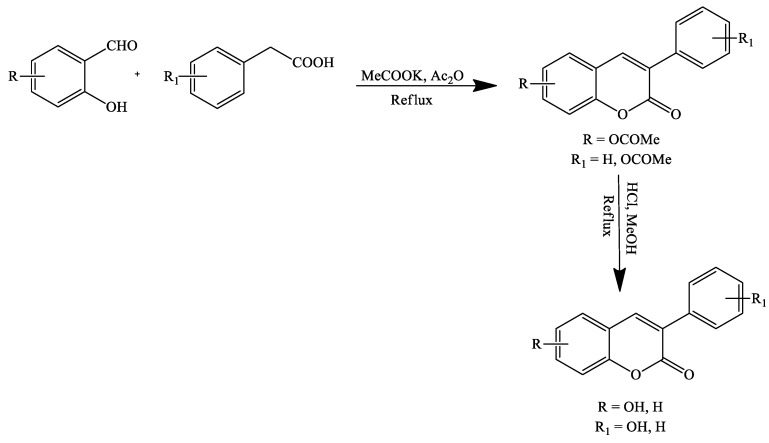
Efficient two-step synthesis of hydroxylated 3-phenylcoumarins.

Matos et al. developed efficient two-step synthesis of hydroxylated 3-phenylcoumarins [50]. Initially, acetoxy-3-phenylcoumarins were synthesized in the reaction of salicylaldehydes and phenylacetic acid in the presence of anhydrous potassium acetate (CH_3_CO_2_K) and acetic anhydride. Obtained products were hydroxylated under reflux in the presence of aqueous hydrochloric acid and MeOH (Scheme 27).

**Scheme 28:**
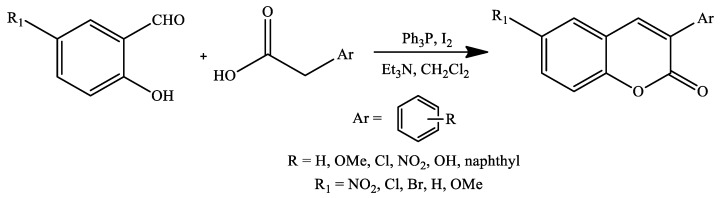
One-pot two-step synthesis of 3-aryl coumarins in the presence of Ph_3_P/I_2_-Et_3_N.

Phakhodee and co-workers reported one-pot two-step synthesis of 3-aryl coumarins between aryl acetic acids and 2-hydroxybenzaladehydes in the presence of Ph_3_P/I_2_-Et_3_N (Scheme 28) [87]. Model reaction of salicylaldehyde and 4-methoxyphenylacetic acid was performed changing the type of the base (Et_3_N, DMAP, imidazole, DABCO and NNM) and solvent (DCM, toluene, DMF and MeCN). Best yield of 98% was obtained when DCM as solvent and triethylamine as base were used. Yields of obtained products under that reaction conditions were in the range from 41 to 98%.

**Scheme 29:**
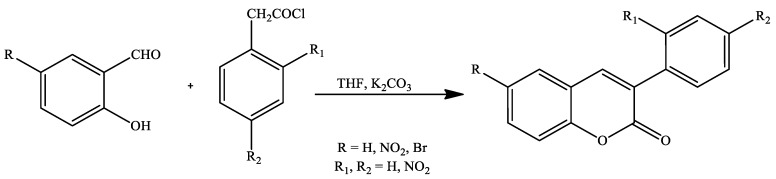
Ultrasound assisted synthesis of 3-phenylcoumarin derivatives in presence of tetrahydrofuran and K_2_CO_3_.

Sripathi and Logeeswari reported synthesis of 3-aryl lcoumarin derivatives using ultrasonic irradiation [88]. 3-Phenylcoumarins were synthesized from salicylaldehydes and phenyl acetyl chloride with the addition of tetrahydrofuran (THF) and K_2_CO_3_ (Scheme 29). The yields of obtained 3-phenylcoumarins were ranged from 7 to 98%.

**Scheme 30:**
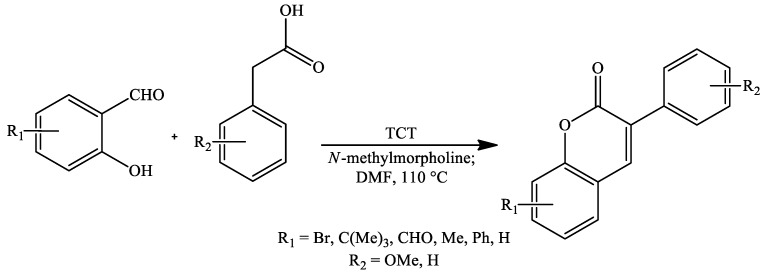
Efficient synthesis of 3-aryl coumarin derivatives in presence of N-methylmorpholine.

Sashidhara et al. have reported an efficient synthesis of 3-aryl coumarin derivatives through reaction of 2-hydroxybenzaldehyde and phenylacetic acid derivatives (Scheme 30) [89]. This reaction was conducted in the presence of cyanuric chloride (2,4,6-trichloro-1,3,5-triazine, TCT). Reaction conditions were optimized by changing different parameters (base types and molar ratios, reaction times, temperatures and solvents). The best reaction conditions were proven to be N-methylmorpholine at 1.5 mmol in DMF at temperature of 110 °C affording product yield of 95%. After the optimization, reactions of substituted 2-hydroxybenzaldehydes and diverse phenylacetic acids were performed in order to obtain corresponding 3-aryl coumarins. 

**Scheme 31:**
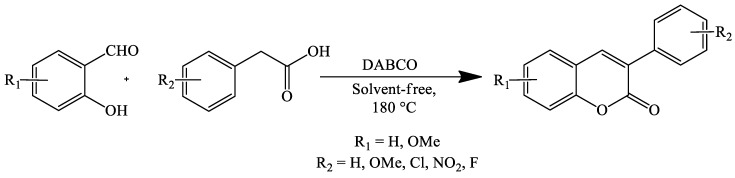
Solvent-free synthesis of 3-aryl coumarins in the presence of DABCO as the catalyst.

Rahmani-Nezhad et al. conducted solvent-free reaction between substituted salicylaldehydes and various phenylacetic acids in the presence of 1,4-diazabicyclo[2.2.2] octane (DABCO) to afford 3-aryl coumarins (Scheme 31) [90]. Optimization of the reaction conditions was performed changing solvents (EtOH, MeOH, toluene, THF, DMF and solvent-free), temperature and DABCO amount. The best yield of 90% was obtained at 180 °C under solvent-free condition. 3-Aryl coumarins were synthesized in good to excellent yields (61–91%).

**Scheme 32:**
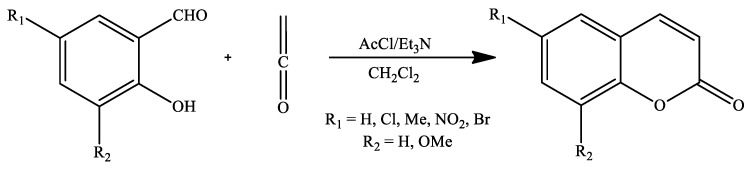
Synthesis of substituted coumarin derivatives from salicylaldehydes and ketene.

Chandrasekhar and Kumar reported synthesis of coumarin derivatives from different salicylaldehydes and ketene (Scheme 32) [91]. All reactions were performed in the presence of trimethylamine, acetyl chloride and DCM. Crude products were obtained in yields 58–72%.

**Scheme 33:**
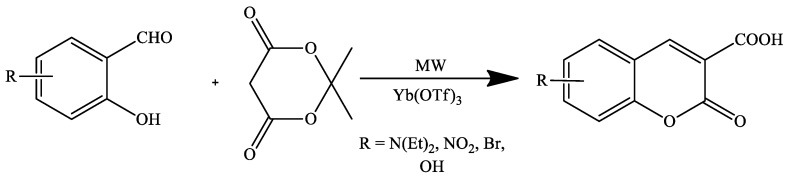
Solvent-free synthesis of coumarin-3-carboxylic acids under microwave irradiation.

Fiorito et al. developed method for the synthesis of coumarin-3-carboxylic acids [92]. This method involved a reaction of substituted salicylaldehydes and Meldrum’s acid (Scheme 33). Reactions were performed under microwave irradiation and solvent-free conditions in the presence of ytterbium triflate (Yb(OTf)_3_). Compounds are obtained in excellent yields (93–98%).

**Scheme 34:**
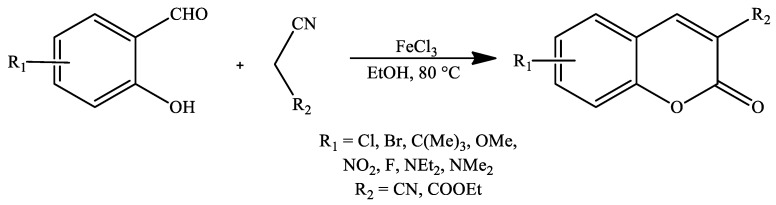
Synthesis of 3-substituted coumarins catalyzed by iron (III) chloride.

Synthesis of 3-substituted coumarins catalyzed by iron(III) chloride was reported by He et al. [34]. Model reaction of salicylaldehydes and malononitrile was performed in order to determine the best reaction conditions. Among several solvents in which reaction was performed (MeOH, EtOH, MeCN, THF, DMF, H_2_O, toluene and DMSO), EtOH is chosen as the best, due to the highest obtained yield (72%). Further, reactions between substituted salicylaldehydes and malononitrile or ethyl 2-cyanoacetate were conducted (Scheme 34). Yields obtained with ethyl 2-cyanoacetate were higher than with malonoitrile.

**Scheme 35:**
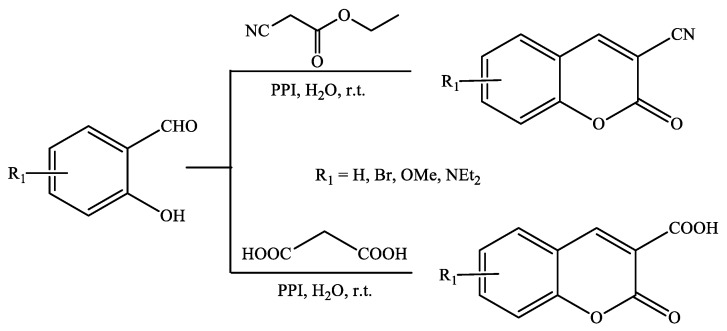
Synthesis of 3-substituted coumarins in the presence of potassium phtalamide.

Kiyani and Daroonkala synthesized 3-substituted coumarins in the presence of potassium phtalamide (PPI) [93]. Initially, model reaction was performed in order to optimize reaction conditions. Reaction of salicylaldehydes and malononitrile was performed in the presence of different catalysts (sodium ascorbate, sodium citrate, sodium tetraborate, K_2_CO_3_, Na_2_CO_3_ and PPI), solvents (EtOH, MeOH, MeCN, CH_2_Cl_2_ and water) and under different temperatures (room temperature, 50, 75 °C and reflux). The highest yield of 92% was obtained at room temperature in the presence of PPI and water. Under optimized conditions reactions of substituted salicylaldehydes and malonic acid or ethyl 2-cyanoacetate were conducted (Scheme 35). Yields of obtained products were in the range from 87 to 93%.

**Scheme 36:**
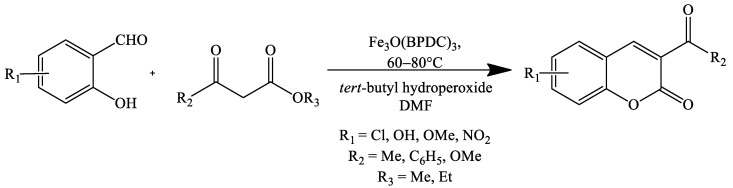
Synthesis of substituted coumarins catalyzed by Fe_3_O(BPDC)_3_.

Synthesis of several coumarins catalyzed by Fe_3_O(BPDC)_3_ was reported by Lieu et al. [94]. In order to find the best reaction conditions following parameters were investigated: effect of temperature, solvent, catalyst concentration, reagent molar ratio, oxidant concentration and type, non-grinded and grinded Fe_3_O(BPDC)_3_ and different homogenous catalysts. Under optimized reaction conditions synthesis of coumarins between various substituted salicylaldehydes and activated methylene compounds were performed as is shown in Scheme 36.

**Scheme 37:**
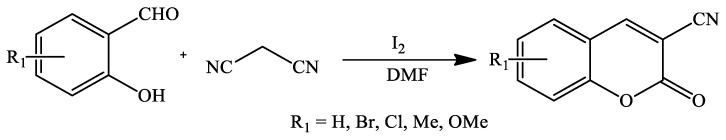
Synthesis of 3-cyanocoumarins performed under heating and microwave conditions.

Sharma and Makrandi conducted synthesis of 3-cyanocoumarins under heating conditions and microwave irradiation [95]. Reactions of 2-hydroxybenzaldehydes with malononitrile in the presence of iodine as a catalyst (Scheme 37) were performed in DMF. Yields of the obtained 3-cyanocoumarins synthesized under heating and microwave conditions were in the range 80–92% and 85–95%, respectively.

In Table 1, the most reported synthesis methods of coumarin derivatives were shown. Substituted 2-oxo-2*H*-chromene-3-carboxylic acids were synthesized in good to excellent yield (73–99%) in all reported methods (Table 1). The highest yields 93–98% were obtained in a solvent-free method under microwave irradiation in the presence of Yb(OTf)_3._ All mentioned methods for the synthesis of 2-oxo-2*H*-chromene-3-carboxylic acids can be considered as green, except the method performed in the presence of sodium azide, which has toxic properties. Due to the negligible differences in the obtained yields, this method can be easily replaced by methods mentioned above. For the synthesis of substituted 2-oxo-2*H*-chromene-3-carbonitriles, 10 methods are listed in Table 1. The yield of the obtained products were 49–98%. Two methods should be pointed out as eco-friendly methods, synthesis performed in deep eutectic solvent and synthesis in water in the presence of choline chloride as a catalyst. Deep eutectic solvents are considered as green solvents, which have a dual role (solvent and catalyst) and choline chloride is a biodegradable catalyst. In both methods, obtained yields were good to excellent. High yields in range from 85 to 98% were obtained in a method reported by Augustine and co-workers [75]. However, high temperature application and usage of propylphosphonic anhydride and trimethylamine, both having toxic properties, should be replaced by a method with mild reaction conditions. In Table 1 four methods are listed for synthesis of substituted 3-acetyl-2*H*-chromen-2-ones. The highest yields (92–96%) were obtained in an environmentally benign method under solvent-free conditions and ultrasound irradiation. Among three mentioned methods for the synthesis of substituted methyl 2-oxo-2*H*-chromene-3-carboxylates, eco-friendly method in water, with choline chloride as catalyst, is proven to be the best. Substituted ethyl 2-oxo-2H-chromene-3-carboxylates were synthesized by various methods (Table 1) with the yields from 25 to 95%. High yields (91–92%) were obtained in a reaction with choline chloride in water and in a solvent-free reaction with MgFe_2_O_4_ nanoparticles as a catalyst (88–93%). Piperidine, which has toxic properties, was applied in three methods in the synthesis of substituted ethyl 2-oxo-2*H*-chromene-3-carboxylates. Obtained yields in these reactions were lower than in mentioned methods with mild conditions.

## 3. Coumarin Derivatives Synthesized from Phenols

**Scheme 38:**
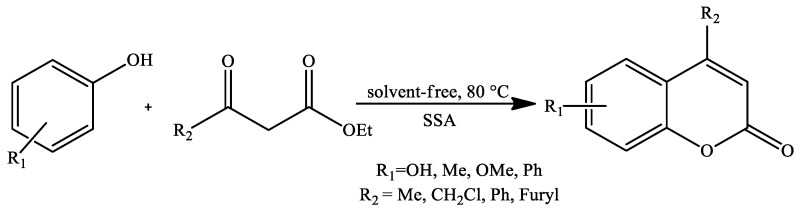
Synthesis of coumarin derivatives in presence of starch sulfuric acid.

In order to synthesize coumarins derivatives, a Pechmann condensation of phenols with β-ketoesters in the presence of starch sulfuric acid (SSA) as a catalyst, is performed by Rezaei et al. [96]. Model reaction of resorcinol and ethyl acetoacetate was performed under solvent-free conditions in the presence of SSA at 80 °C. Different coumarin derivatives were obtained in the reactions between various substituted phenols and β-ketoesters (ethyl acetoacetate, ethyl 4-chloroacetoacetate, ethyl benzoylacetate, ethyl furanoacetate; Scheme 38).

**Scheme 39:**
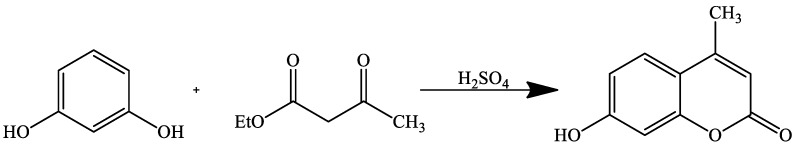
Coumarins synthesis via Pechmann condensation with sulfuric acid as a catalyst.

Pornsatitworakul et al. synthesized coumarin via Pechmann condensation between resorcinol and ethyl acetoacetate (Scheme 39) with a few drops of concentrated sulfuric acid as a catalyst [97]. Yields of the obtained coumarin was 86%.

**Scheme 40:**
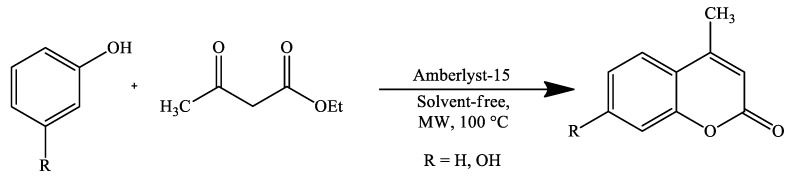
Solvent-free synthesis of 4-methylcoumarins under microwave irradiation.

Bouasla et al. developed solvent-free synthesis of 7-hydroxy-4-methylcoumarin and 4-methylcoumarin under microwave irradiation with different catalysts (Amberlyst-15, zeolite β and sulfonic acid functionalized hybrid silica) [98]. In the model reaction between resorcinol and ethyl acetoacetate via Pechmann condensation, Amberlyst-15 was proven to be the best catalyst giving the yield of 97%. 4-Methylcoumarin was synthesized in reaction of phenol and ethyl acetoacetate (Scheme 40) affording the highest yield of 43% in the presence of Amberlyst-15.

**Scheme 41:**
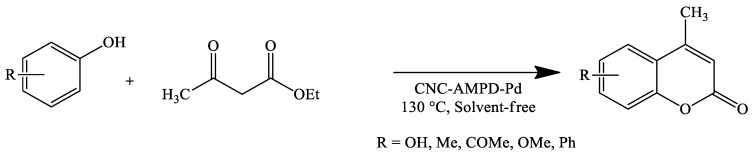
Solvent-free Pechmann condensation of coumarin derivatives in the presence of cellulose nanocrystal supported palladium nanoparticles.

Solvent-free Pechmann condensation of coumarin derivatives in the presence of cellulose nanocrystal supported palladium nanoparticles (CNC-AMPD-Pd) was developed by Mirosanloo et al. [99]. Reaction of resorcinol and ethyl acetoacetate was chosen to be a model reaction. In order to achieve the best reaction conditions different solvents and temperatures were applied. The highest yield of 96% was obtained in a solvent-free reaction at 130 °C. Coumarin derivatives were synthesized in a reaction of various phenols and ethyl acetoacetate (Scheme 41) giving yields from 45 to 97%.

**Scheme 42:**
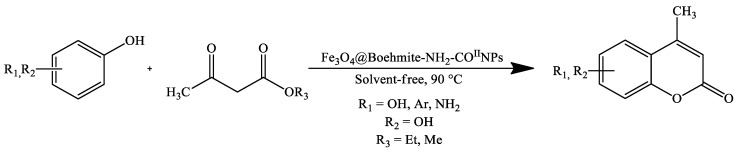
Synthesis of coumarin derivatives in the presence of magnetic-core-shell-like Fe_3_O_4_@Boehmite-NH_2_-Co^II^ NPs.

Synthesis of coumarin derivatives in the presence of magnetic-core-shell-like Fe_3_O_4_@Boehmite-NH_2_-Co^II^ NPs was reported by Pakdel et al. [100]. Reaction conditions of Pechmann condensation were studied on the reaction between resorcinol and ethyl acetoacetate. Amount of the catalyst, solvent and temperature were changed in order to obtain optimal reaction conditions. Reactions were also carried out with various refluxing solvent (MeOH, EtOH, DMF, MeCN and H_2_O). The highest yield of 90% was obtained in a solvent-free reaction at 90 °C with 6.6 mol% of Fe_3_O_4_@Boehmite-NH_2_-Co^II^. Further, different phenols and β-ketoesters (ethyl and methyl acetoacetate) were coupling to afford coumarin derivatives (Scheme 42).

**Scheme 43:**
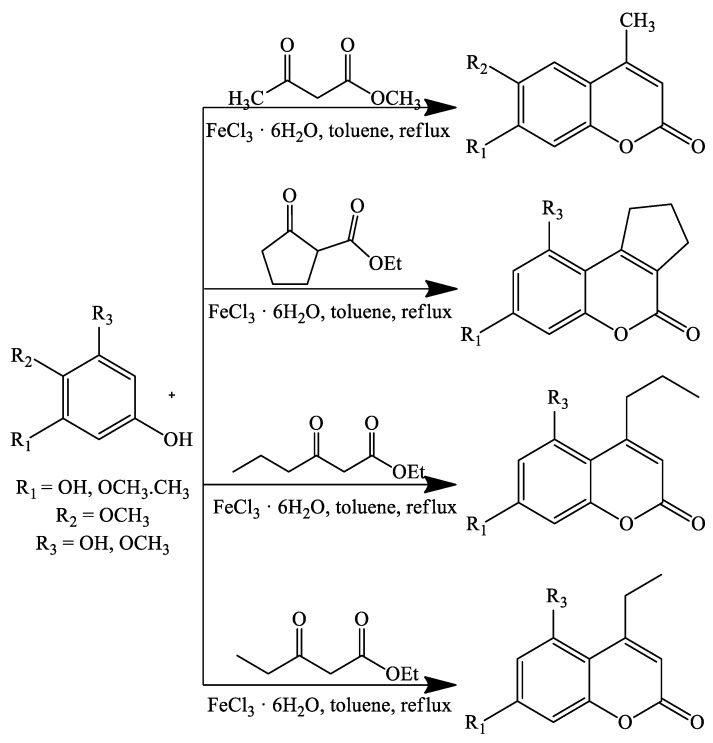
Condensation reaction of phenols and β-ketoesters catalyzed by FeCl_3_·6H_2_O.

The Pechmann method has been used for the synthesis of coumarin derivatives in the presence of FeCl_3_·6H_2_O [101]. The feasibility of iron salt catalysts were observed in condensation reaction of resorcinol and methyl acetoacetate. Yield of 92% was achieved in a reaction with 10 mol% FeCl_3_·6H_2_O in toluene under reflux for 16 h. Various coumarins were obtained in a reaction of different phenols and β-ketoesters under optimized conditions (Scheme 43).

**Scheme 44:**
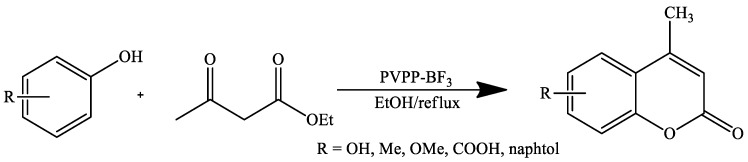
Synthesis of 4-methyl coumarin derivatives via Pechmann condensation.

4-Methyl coumarins were synthesized via Pechmann condensation in the presence of polyvinylpolypyrrolidone-bound boron trifluoride (PVPP-BF_3_) [102]. Reaction of phenols and ethyl acetoacetate was performed in EtOH with PVPP- BF_3_ as catalyst under reflux conditions (Scheme 44). Pure coumarin crystals were obtained in yield of 72–96%.

**Scheme 45:**
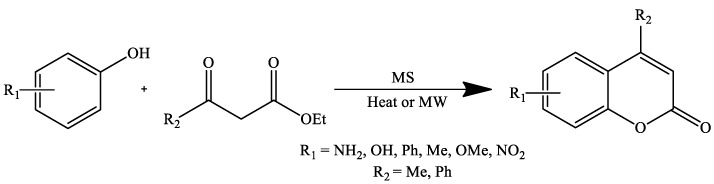
Solvent-free synthesis of coumarin derivatives with meglumine sulfate as a catalyst.

Moradi and co-workers performed one-pot solvent-free synthesis of coumarin derivatives with meglumine sulfate (MS) as a catalyst [103]. For comparison, syntheses were conducted under microwave and thermal conditions. As a model reaction Pechmann condensation of resorcinol and ethyl acetoacetate was performed in order to optimize reaction conditions like temperature, time of reaction and catalyst amount. The best reaction conditions were used in further syntheses between ethyl acetoacetate and different substituted phenols, which are carried out under thermal and microwave conditions (Scheme 45). Both conditions gave high to excellent yield (88–93%), but in microwave synthesis reaction time was shorter.

**Scheme 46:**
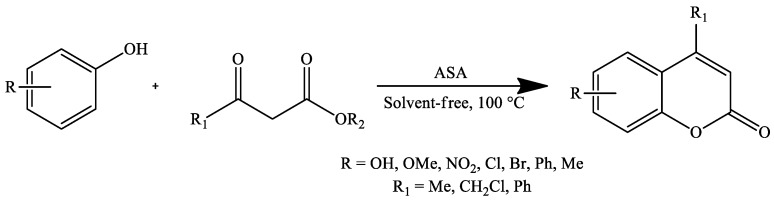
Alumina sulfuric acid catalyzed reaction between phenolic compounds and β-ketoesters.

Amoozadeh et al. reported solvent-free Pechmann condensation for coumarin derivatives synthesis using alumina sulfuric acid (ASA) as reusable catalyst [104]. Model reaction of resorcinol and ethyl acetoacetate was performed in the presence of different alumina and alumina supported acids and the best yield was obtained in a reaction catalyzed by ASA. Further, various coumarin derivatives were prepared in reaction between different phenolic compounds and β-ketoesters (Scheme 46). Yield of the obtained products were in the range 25–99%.

**Scheme 47:**
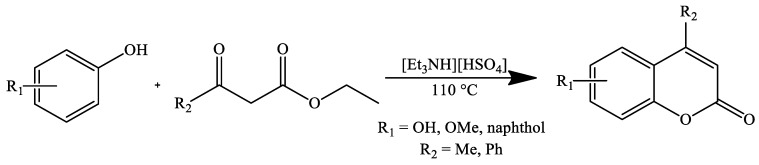
Pechmann synthesis of coumarins in the presence of triethylammonium hydrogen sulfate as a catalyst.

The efficient Pechmann synthesis of coumarins, in the presence of triethylammonium hydrogen sulfate as a catalyst, was performed by Karimi-Jabei et al. [105]. Optimization reaction between resorcinol and ethyl acetoacetate was conducted changing solvents (EtOH, MeOH, CHCl_3_, MeCN and solvent-free) and temperature (room temperature, 50 °C, 110 °C and 130 °C). Temperature of 110 °C and solvent-free condition gave yield of 92%. Various coumarins were obtained in reactions of substituted phenols and β-ketoesters (Scheme 47). Coumarin derivatives were obtained in good to excellent yields (83–95%).

**Scheme 48:**
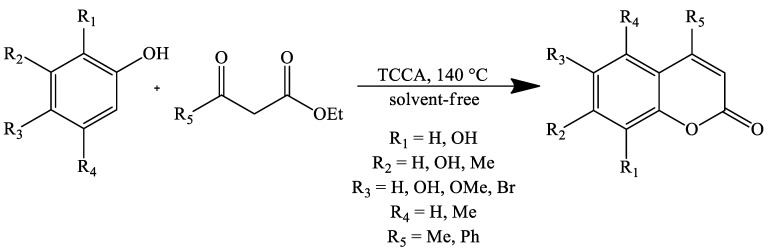
Synthesis of coumarin derivatives in the presence 1,3,5-trichloroisocyanuric acid.

Synthesis of different coumarin derivatives in the presence of TCCA (1,3,5-trichloroisocyanuric acid) via Pechmann condensation was performed by Hojati and Hadadnia [106]. Model reaction of resorcinol and ethyl acetoacetate was conducted in order to find the best reaction conditions. Influence of the catalyst amount, substrates molar ratios, temperature and solvent type was examined. Yield of 97% was achieved at 140 °C under solvent-free conditions with optimum amounts of 1:1.2:0.1 for resorcinol, ethyl acetoacetate and TCCA. Series of phenols were reacted with β-ketoesters under optimized conditions to obtain corresponding coumarins (Scheme 48).

**Scheme 49:**
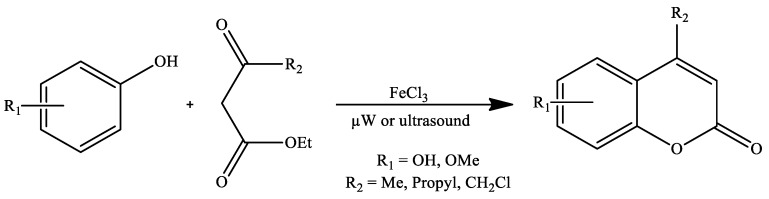
Ultrasound assisted solvent-free synthesis of 4-substituted coumarins.

Prousis et al. performed microwave and ultrasound assisted solvent-free synthesis of 4-substituted coumarins [107]. Model reaction between phloroglucinol and ethyl acetoacetate in the presence of FeCl_3_ were carried out in order to obtain the optimal reaction conditions. The optimal reaction conditions for microwave irradiation (100 °C/150 W, 10 min) and the catalyst amount (10 mol%) were determined. Parameters for ultrasound irradiation were as follows: 20 kHz, nominal power 130 W, 18 min. Reactions of the phenol, β-ketoester and anhydrous FeCl_3_ (Scheme 49) under microwave and ultrasound irradiation were also performed under conventional heating method at 70 °C in order to compare methods efficiency. It turned out that ultrasound afforded the best yield of the products.

**Scheme 50:**
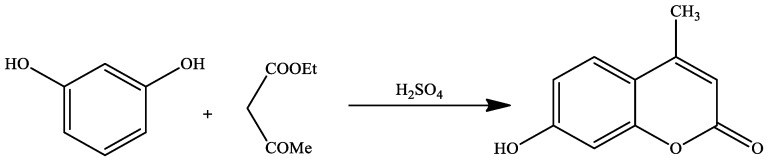
Synthesis of 7-hydroxy-4-methyl coumarin in presence of H_2_SO_4_ under ultrasonic irradiation.

Silveira Pinto and Souza performed a synthesis of 7-hydroxy-4-methylcoumarin, between resorcinol and ethylacetoacetate (Scheme 50) [70]. Reaction was conducted in presence of H_2_SO_4_ (70%) under ultrasonic irradiation. Yield of obtained product was 87%.

**Scheme 51:**
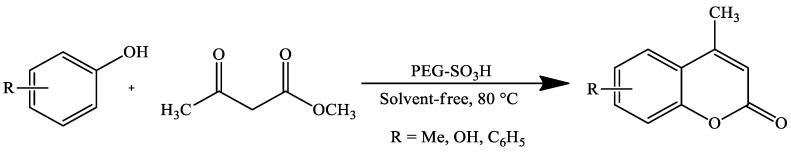
Synthesis of 4-methyl coumarins in PEG-SO_3_H catalyzed reaction.

Nazeruddin et al. have reported an efficient synthesis of coumarins derivatives in PEG-SO_3_H catalyzed reaction [108]. Solvent-free syntheses was performed at 80 °C, between substituted phenols and dicarbonyl compounds with PEG-SO_3_H as a recyclable catalyst (Scheme 51). Products were obtained in yield 78–91%.

**Scheme 52:**
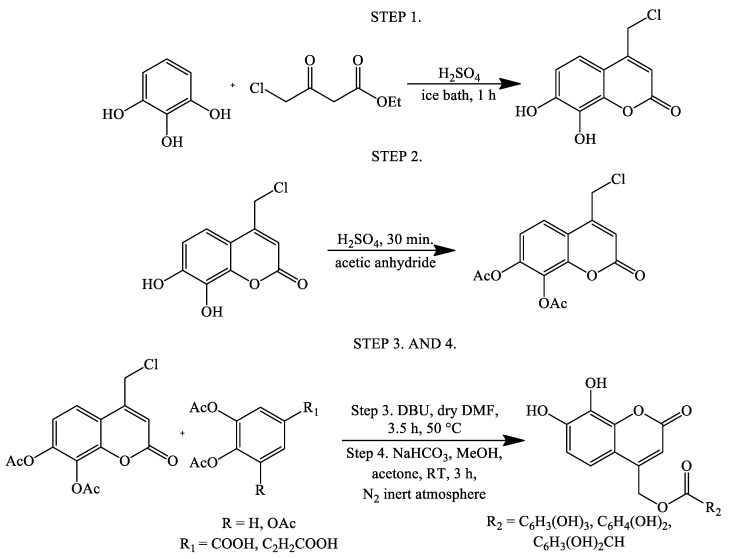
Four-step syntheses of polyphenolic hybrid-coumarins.

Perez-Cruz et al. developed four-step syntheses of polyphenolic hybrid-coumarins [51]. The first step was a Pechmann condensation of pyrogallol and 4-chloroethylacetoacetate, which afforded 4-chloromethyl-7,8-dihydroxycoumarin. Reaction was performed in the presence of H_2_SO_4_ on ice bath for 1 h. In the second step, hydroxyl groups were protected as acyl ester derivatives. In the last two steps hybrid compound formation and deacylation of hydroxyl groups were performed. The entire procedure with appropriate reaction conditions is shown in Scheme 52.

**Scheme 53:**
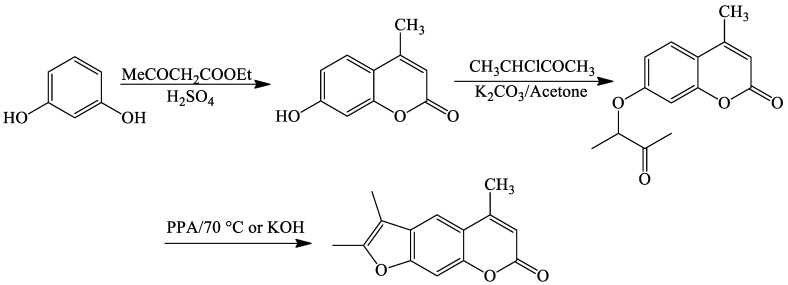
Three-step synthesis of furanocoumarin.

Three-step synthesis of furanocoumarins was investigated by Elgogary et al. [109]. In the first step, Pechmann condensation of resorcinol, quinol and orcinol with ethyl acetoacetate in sulfuric acid was performed in order to obtain hydroxycoumarins. Further, Williamson reaction of hydrocoumarins with 3-chloro-2-butanone was conducted affording keto ethers. The reactions were carried out in refluxing acetone with K_2_CO_3_ as a catalyst. The last step was a cyclization of keto ethers with polyphosphoric acid (PPA) at 70 °C or by heating in alkaline solution (3% KOH). All reaction steps are shown in Scheme 53 on a resorcinol example.

**Scheme 54:**
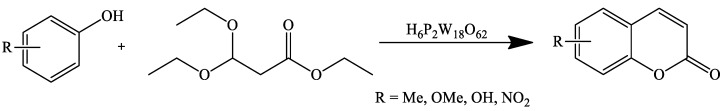
Solvent-free synthesis of substituted coumarins catalyzed by Wells-Dawson heteropolyacid (H_6_P_2_W_18_O_62_).

Sun et al. have developed a method for the of coumarins unsubstituted on the pyranic nucleus, under solvent-free conditions (Scheme 54) [110]. First, model reaction of 2-methyl-3-hydroxyphenol and ethyl 3,3-diethoxypropionate were performed in order to choose efficient catalyst and to optimize reaction conditions. The best catalyst was proven to be Wells-Dawson heteropolyacid (H_6_P_2_W_18_O_62_). The highest yield of coumarin was obtained under the following conditions: reaction time of 3 h, temperature of 90 °C, raw material ratio of 1:1.15 and 0.25 mmol of catalyst. Further reactions were carried out between various phenol derivatives and ethyl 3,3-diethoxypropionate afforded products in poor to high yields (< 5–90%).

**Scheme 55:**
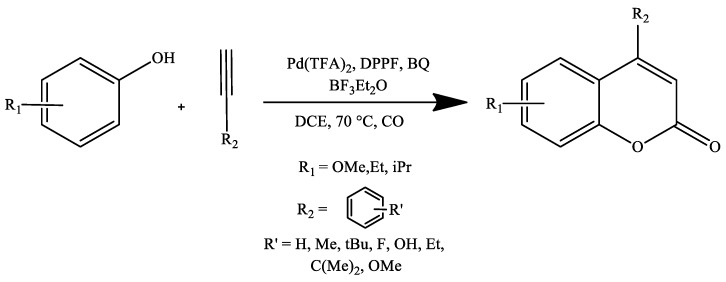
Palladium catalyzed synthesis of coumarin derivatives.

Palladium catalyzed synthesis of various coumarins was reported by Zhu and Wu [111]. Reaction of 4-methoxyphenol and phenylacetylene was initially performed in order to find the best reaction conditions. Different ligands, oxidants and additives were employed in reaction to achieve the best product yield. Different substituted coumarins were obtained between phenols and alkynes under optimized conditions as is shown in Scheme 55.

In Table 2 methods for the most synthesized coumarins from phenols were presented. In all listed methods, the synthesis of substituted 4-methyl-2I-chromen-2-ones reactions were performed under solvent-free conditions, except two methods, that used ethanol and toluene as solvent (Table 2). The highest yields (88–93%) were obtained in a solvent-free method, with meglumine sulfate as a catalyst, under microwave irradiation. In addition, meglumine sulfate can be considered as an eco-friendly catalyst. Some of the used catalysts, such as the cellulose nanocrystal supported palladium nanoparticles (CNC-AMPD-Pd), PEG-SO_3_H and alumina sulfuric acid can be recycled, which provides economic and environmental advantages. Reactions with catalysts, as H_2_SO_4_ and the solvent, as toluene, should be replaced with the synthetic methods with mild conditions. All listed substituted 4-phenyl-2*H*-chromen-2-ones were synthesized under solvent-free conditions. The highest yields were obtained under the microwave irradiation (88–92%) in the presence of meglumine sulfate as a catalyst. Substituted 4-(chloromethyl)-2*H*-chromen-2-ones were synthesized with five methods mentioned in the Table 2. Yields in the range from 88 to 96% were obtained under solvent-free conditions at 100 °C in the presence of alumina sulfuric acid as a catalyst. The lowest yield (68%) was obtained in the reaction with FeCl_3_ under microwave irradiation at 100 °C.

## 4. Coumarin Derivatives Synthesized from Ketones

**Scheme 56:**
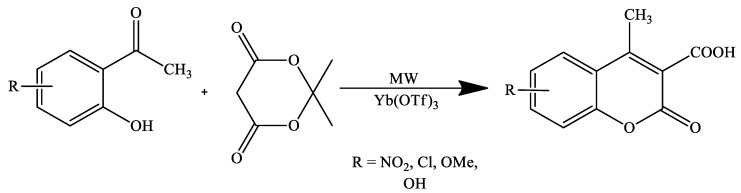
Synthesis of coumarin-3-carboxylic acids under microwave irradiation.

Fiorito et al. [92] synthesized coumarin-3-carboxylic acids under microwave irradiation. The reaction was performed under the solvent-free conditions in the presence of ytterbium triflate (Yb(OTf)_3_), between substituted 2-hydroxyacetophenones and Meldrum’s acid (Scheme 56). Yields of the obtained compounds were in the range from 92 to 98%.

**Scheme 57:**
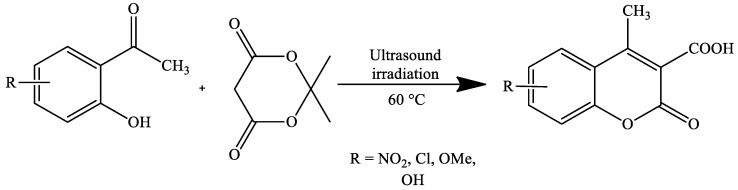
Synthesis of coumarin derivatives via Knoevenagel condensation in green solvents.

Knoevenagel condensation of 2-hydroxyacetophenones and Meldrum’s acid was performed in green solvents (edible fruit and vegetable juices, liqueurs and wastewaters), under ultrasound irradiation at 60 °C by Fiorito et al. (Scheme 57) [72]. Yields were in the range from 94 to 99% and highest conversion was observed in limoncello.

**Scheme 58:**
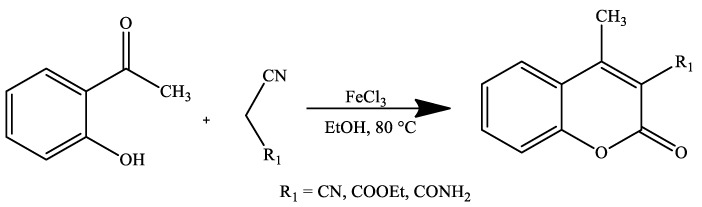
Synthesis of 3,4-disubstituted coumarins in presence of FeCl_3._

He et al. reported synthesis of 3,4-disubstituted coumarins from 2-hydroxy acetophenone and 2-cyanoacetamide, malononitrile and ethyl 2-cyanoacetate (Scheme 58) [34]. Reaction was performed in presence of FeCl_3_ at 80 °C. Yields of obtained coumarins were 41–63%.

**Scheme 59:**
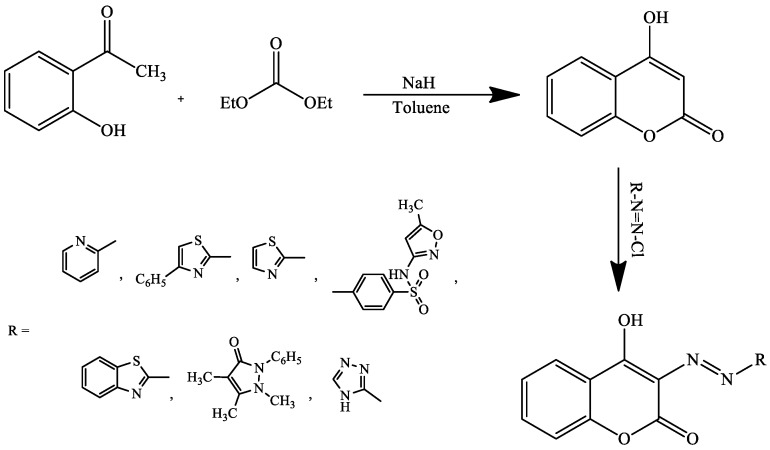
Two-step synthesis of 4-hydroxy-3-(hetero aryl Azo) coumarins.

Sahoo et al. reported synthesis of 4-hydroxy-3-(hetero aryl Azo) coumarins [43]. Initially, 4-hydroxy coumarin was synthesized via Claisen condensation of 2-hydroxy acetophenone and diethyl carbonate, in the presence of sodium hydride in toluene. Further, a coupling of 4-hydroxy coumarins with diazonium salt yielded various hetero aryl amine derivatives (Scheme 59). Products were obtained in yields from 55–82%.

**Scheme 60:**
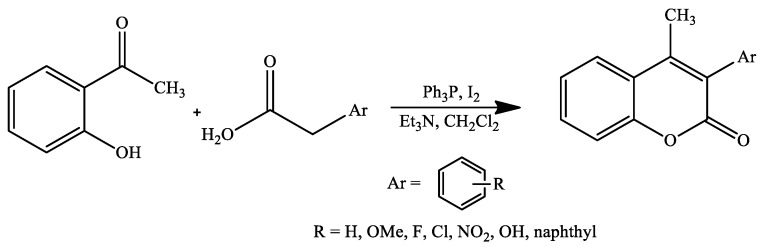
One-pot two-step synthesis of 3-aryl-4-methylcoumarins.

Phakhodee and co-workers disclosed the one-pot two-step synthesis of 3-aryl-4-methylcoumarins [87]. Reactions were mediated by Ph_3_P/I_2_-Et_3_N. In model reaction type of base (Et_3_N, DMAP, imidazole, DABCO and NNM) and solvent (DCM, toluene, DMF and MeCN) were changed in order to obtain the best reaction conditions. Salicylaldehyde and 4-methoxy phenylacetic acid were used as a model substrates. All reactions were mediated by Ph_3_P/I_2_ and carried out at room temperature. The highest yield of coumarins was obtained when DCM as a solvent and triethylamine as a base were used. Series of coumarins was synthesized using aryl acetic acids and 2′-hydroxyacetophenones (Scheme 60) under optimized reaction conditions and obtained in good to excellent yields (52–89%).

**Scheme 61:**
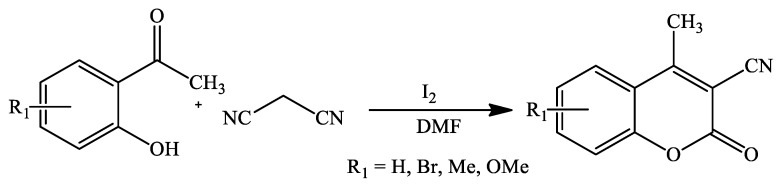
Synthesis of 3-cyano-4-methylcoumarions in presence of iodine as a catalyst. Coumarin derivatives synthesized from carboxylic acids.

Synthesis of 3-cyano-4-methylcoumarions was conducted under heating and microwave conditions by Sharma and Makrandi [95]. Reactions of 2-hydroxyacetophenones with malononitrile in presence of iodine as catalyst (Scheme 61) were performed in DMF. Yields of the obtained coumarin derivatives were similar in both methods (heating (85–90%) and microwave (87–95%)), but reaction time was significantly shorter under microwave conditions.

**Scheme 62:**
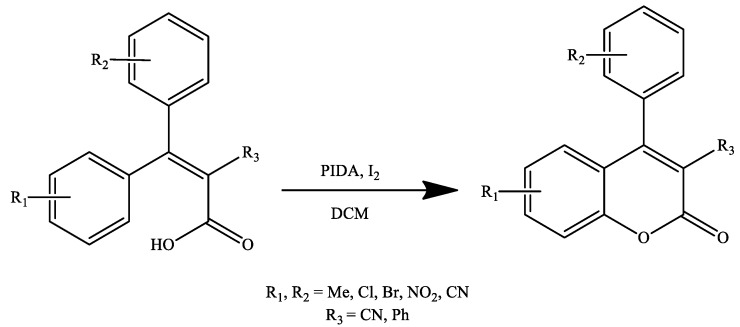
Synthesis of coumarin derivatives performed by Li et al.

Li et al. developed a method for the synthesis of various substituted coumarins [112]. Initially, optimization of the reaction conditions was conducted with phenylacrylic acid, in the presence of different solvents (TFA, DCM, toluene, DMF, MeCN and EtOH), additives (BF_3_·Et_2_O, TFA, LiBr and I_2_) and oxidants (PIFA and PIDA). Further, reaction of diaryl acrylic acids was carried out under the optimized conditions (DCM, I_2_ and PIDA; Scheme 62). A series of coumarins was obtained in good to excellent yields (41–92%).

**Scheme 63:**
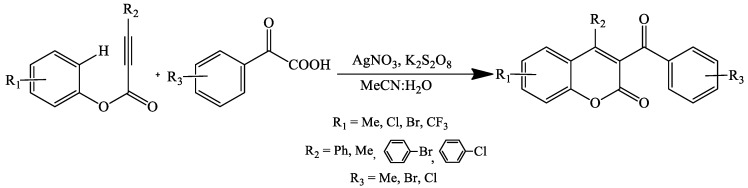
Radical cyclization of α-keto acids and alkynoates in coumarin derivatives synthesis.

Yan et al. developed a method that involved reaction of *α*-keto acids and alkynoates [113]. Model reaction of 3-phenylpropiolate and 2-oxo-2-phenylacetic acid was performed under different conditions, changing solvents (MeCN:H_2_O, H_2_O and DMF:H_2_O), oxidants (Na_2_S_2_O_8_, (NH_4_)_2_S_2_O_8_, TBHP, O_2_ and K_2_S_2_O_8_) and catalysts (none, AgNO_3_, Ag_2_O, Ag_2_CO_3_ and AgOAc). The highest yield of 75% was obtained under the following reaction conditions: AgNO_3_, K_2_S_2_O_8_ and MeCN:H_2_O. Further, coumarin derivatives were synthesized via radical cyclization as is shown in Scheme 63. Yields of the obtained products were 40–76%.

**Scheme 64:**
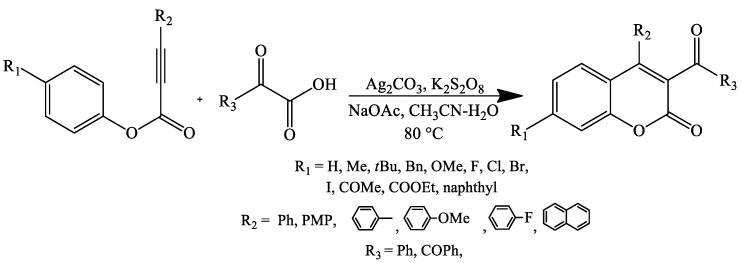
Silver-promoted decarboxylative annulation of 3-acylcoumarins.

3-Acylcoumarins were obtained via silver-promoted decarboxylative annulation by Liu et al. [114]. Initial studies were conducted in order to determine optimal reaction conditions. Reaction of alkynoate and 2-phenyl-2-oxoacetic acid was performed in presence of different catalysts (Ag_2_CO_3_ and AgNO_3_), oxidants (K_2_S_2_O_8_, (NH_4_)_2_S_2_O_8_, oxone, PhI(OAc)_2_ and TBHP), additives (Na_2_CO_3_, NaOAc, KHCO_3_ and KOH) and solvents (DMF, MeCN, DMF-H_2_O and MeCN-H_2_O). Highest yield of 74% was obtained in the presence of Ag_2_CO_3_, K_2_S_2_O_8_, NaOAc and MeCN-H_2_O. A series of 3-acylcoumarins was obtained in a reaction of substituted alkynoates and 2-oxoacetic acids (Scheme 64). Yields of obtained products were from 45 to 78%.

## 5. Conclusions

The medicinal properties of coumarin have been discovered due to their presence in different medicinal plants. Coumarin isolation process from those plants is time consuming and expensive and only small amounts of desired compounds can be obtained Therefore, the synthesis of these derivatives is a faster and in some cases “greener” way to obtain the desired compounds. Coumarins possess various biological activities and have a positive effect on human health. The purpose of this review was to present different methods of coumarin synthesis, both conventional and green ones. Syntheses from different starting compounds like aldehydes, phenols, ketones and carboxylic acids were described, in order to provide a deeper insight into the possibilities of their formation and offer the researchers dealing with this subject a range of different synthetic approaches. All of these syntheses have different reaction conditions. Different techniques such as heating, microwave and ultrasound irradiation were employed in coumarin synthesis. In addition, various solvents and catalysts were used in order to obtain coumarin derivatives in high yields. Some of catalysts and solvents are harmful and some have green character. In some cases it has been shown that compounds obtained by green methods have higher yields than compound obtained with conventional methods. It was also observed that higher yields were obtained for components synthesized from aldehydes. As it tends to reduce environmental pollution, there is an increasing interest in developing green methods and reducing the use of harmful compounds. During synthesis, it is important to reduce energy consumption, to avoid harmful substances and to obtain pure compounds in high yields. This comparison of reaction conditions and obtained yields of compounds will be useful to scientists in this field of work to develop new efficient methods.

## Figures and Tables

**Table 1:** Comparison of methods for the synthesis of coumarin derivatives from aldehydes.

Reaction Conditions	Solvent	Catalyst	Yields (%)	Reference
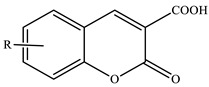 Substituted 2-oxo-2*H*-chromene-3-carboxylic acids				
Microwave irradiation	Solvent-free	Yb(OTf)_3_	93–98	[92]
Stirring, RT	Water	Potassium phtalamide (PPI)	87–90	[93]
Stirring, RT	Water	K_2_CO_3_	73–93	[69]
		NaN_3_	78–99	
Ultrasound irradiation	Water	No-catalyst	80	[70]
Reflux			95	
Stirring, RT	Lemon, pomegranate, grapefruit, carrot, tomato, kiwi and limoncello juice, vinegar, olive mil and buttermilk waste water	No-catalyst	91–99	[72]
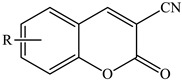 Substituted 2-oxo-2*H*-chromene-3-carbonitriles				
Stirring, 25–30 °C	Water	Choline chloride	79–87	[66]
Stirring, RT	Water	Potassium phtalamide (PPI)	89–93	[93]
Ultrasound irradiation	Ethanol	Piperidine	49	[70]
Reflux			50	
Stirring, 35–40 °C	Ethanol	PhI(OAc)_2_	80–92	[71]
Stirring, 80 °C	Ethanol	FeCl_3_	72–93 *	[34]
Stirring, 80 °C	Deep eutectic solvent	Deep eutectic solvent	73–92	[65]
Reflux	Dimethylformamide	I_2_	80–92	[95]
Microwave irradiation			85–95	
Stirring, 120 °C	Butyl acetate	Propylphosphonic anhydride (T3P), trimethylamine (TEA)	85–98	[75]
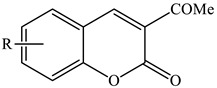 Substituted 3-acetyl-2H-chromen-2-ones				
Ultrasound irradiation, 45 °C	Solvent-free	MgFe_2_O_4_ nanoparticles	92–96	[73]
Stirring, 25–30 °C	Water	Choline chloride	90	[66]
Stirring, 35–40 °C	Ethanol	PhI(OAc)_2_	82–92	[71]
Stirring, 60–80 °C, *tert*-butyl hydroperoxide	Dimethylformamide	Fe_3_O(BPDC)_3_	65–96	[94]
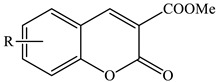 Substituted methyl 2-oxo-2H-chromene-3-carboxylates				
Stirring, 2–30 °C	Water	Choline chloride	87–96	[66]
Stirring, 120 °C	Butyl acetate	Propylphosphonic anhydride (T3P), trimethylamine (TEA)	94	[75]
Stirring, 60–80 °C, *tert*-butyl hydroperoxide	Dimethylformamide	Fe_3_O(BPDC)_3_	28	[94]
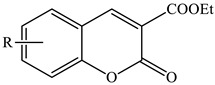 Substituted ethyl 2-oxo-2H-chromene-3-carboxylates				
Ultrasound irradiation, 45 °C	Solvent-free	MgFe_2_O_4_ nanoparticles	88–93	[73]
Stirring, 25–30 °C	Water	Choline chloride	91–92	[66]
Stirring, RT	Ethanol	Piperidine, AcOH	67–83	[68]
Stirring, 35–40 °C	Ethanol	PhI(OAc)_2_	84–92	[71]
Ultrasound irradiation	Ethanol	Piperidine, AcOH	60–88	[70]
Reflux			48–85	
Stirring, 80 °C	Ethanol	FeCl_3_	70–95	[34]
Reflux	Toluene	Piperidine	25–82	[74]

* Some product in traces or no reactions at all.

**Table 2:** Comparison of methods for the synthesis of coumarin derivatives from phenols.

Reaction Conditions	Solvent	Catalyst	Yields (%)	Reference
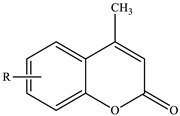 Substituted 4-methyl-2H-chromen-2-ones				
Stirring, 80 °C	Solvent-free	Starch sulfuric acid (SSA)	75–95	[96]
Ultrasound irradiation	Solvent-free	H_2_SO_4_	87	[70]
Stirring	Solvent-free	H_2_SO_4_	86	[97]
Microwave irradiation, 100 °C	Solvent-free	Amberlyst-15	43–97	[98]
Stirring, 130 °C	Solvent-free	Cellulose nanocrystal supported palladium nanoparticles (CNC-AMPD-Pd)	45–97	[99]
Stirring, 90 °C	Solvent-free	Magnetic-core-shell-like Fe_3_O_4_@Boehmite-NH_2_-Co^II^ NPs	60–95 *	[100]
Stirring, 80 °C	Solvent-free	PEG-SO_3_H	78–91	[108]
Stirring, 100 °C	Solvent-free	Meglumine sulfate (MS)	88–92	[103]
Microwave irradiation			88–93	
Stirring, 100 °C	Solvent-free	Alumina sulfuric acid (ASA)	25–99 *	[104]
Stirring, 110 °C	Solvent-free	Triethylammonium hydrogen sulfate	79–94	[105]
Stirring, 140 °C	Solvent-free	TCCA (1,3,5-trichloroisocyanuric acid)	53–98	[106]
Stirring, 70 °C	Solvent-free	FeCl_3_	36–99	[107]
Microwave irradiation, 100 °C			39–99	
Ultrasound irradiation			55–99	
Reflux	Ethanol	Polyvinylpolypyrrolidone-bound boron trifluoride (PVPP-BF_3_)	72–96	[102]
Reflux	Toluene	FeCl_3_·6H_2_O	44–92	[101]
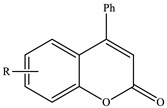 Substituted 4-phenyl-2H-chromen-2-ones				
Stirring, 80 °C	Solvent-free	Starch sulfuric acid (SSA)	78	[96]
Stirring, 100 °C	Solvent-free	Alumina sulfuric acid (ASA)	91	[104]
Heating, 100 °C	Solvent-free	Meglumine sulfate (MS)	88–90	[103]
Microwave irradiation			88–92	
Stirring, 110 °C	Solvent-free	Triethylammonium hydrogen sulfate	85–88	[105]
Stirring, 140 °C	Solvent-free	TCCA (1,3,5-trichloroisocyanuric acid)	50–98	[106]
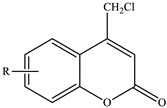 Substituted 4-(chloromethyl)-2H-chromen-2-ones				
Stirring, 80 °C	Solvent-free	Starch sulfuric acid (SSA)	85	[96]
Stirring, 100 °C	Solvent-free	Alumina sulfuric acid (ASA)	88–96	[104]
Stirring, 70 °C	Solvent-free	FeCl_3_	95 *	[107]
Microwave irradiation, 100 °C			68 *	
Ultrasound irradiation			75–96	

* Some product in traces or no reactions at all.

## References

[1] Nikhil B., Shikha B., Anil P., Prakash N.B. (2012). Diverse pharmacological activities of 3-substituted coumarins: A review. Int. Res. J. Pharm..

[2] Kontogiorgis C., Detsi A., Hadjipavlou-Litina D. (2012). Coumarin-based drugs: A patent review (2008–present). Expert Opin. Ther. Pat..

[3] Venugopala K.N., Rashmi V., Odhav B. (2013). Review on Natural Coumarin Lead Compounds for Their Pharmacological Activity. BioMed Res. Int..

[4] Prahadeesh N., Sithambaresan M., Mathiventhan U. (2018). A Study on Hydrogen Peroxide Scavenging Activity and Ferric Reducing Ability of Simple Coumarins. Emerg. Sci. J..

[5] Kulkarni M.V., Kulkarni G.M., Lin C.-H., Sun C.-M. (2006). Recent advances in coumarins and 1-azacoumarins as versatile biodynamic agents. Curr. Med. Chem..

[6] Bairagi S.H., Salaskar P.P., Loke S.D., Surve N.N., Tandel D.V., Dusara M.D. (2012). Medicinal significance of coumarins: A review. Int. J. Pharm. Res..

[7] Thakur A., Singla R., Jaitak V. (2015). Coumarins as anticancer agents: A review on synthetic strategies, mechanism of action and SAR studies. Eur. J. Med. Chem..

[8] Seo W.D., Kim J.Y., Ryu H.W., Kim J.H., Han S.-I., Ra J.-E., Seo K.H., Jang K.C., Lee J.H. (2013). Identification and characterisation of coumarins from the roots of *Angelica dahurica* and their inhibitory effects against cholinesterase. J. Funct. Foods.

[9] Bai Y., Li D., Zhou T., Qin N., Li Z., Yu Z., Hua H. (2016). Coumarins from the roots of *Angelica dahurica* with antioxidant and antiproliferative activities. J. Funct. Foods.

[10] Iranshahi M., Kalategi F., Sahebkar A., Sardashti A., Schneider B. (2010). New sesquiterpene coumarins from the roots of *Ferula flabelliloba*. Pharm. Boil..

[11] Peng W.-W., Zheng Y.-Q., Chen Y.-S., Zhao S.-M., Ji C.-J., Tan N.-H. (2013). Coumarins from roots of *Clausena excavata*. J. Asian Nat. Prod. Res..

[12] Naseri M., Monsef-Esfehani H., Saeidnia S., Dastan D., Gohari A. (2013). Antioxidative Coumarins from the Roots of *Ferulago subvelutina*. Asian J. Chem..

[13] Joshi K.R., Devkota H.P., Yahara S. (2013). Chemical Analysis of Flowers of *Bombax ceiba* from Nepal. Nat. Prod. Commun..

[14] Sukumaran S., Kiruba S., Mahesh M., Nisha S., Miller P.Z., Ben C., Jeeva S. (2011). Phytochemical constituents and antibacterial efficacy of the flowers of *Peltophorum pterocarpum* (DC.) Baker ex Heyne. Asian Pac. J. Trop. Med..

[15] Kicel A., Wolbis M. (2012). Coumarins from the flowers of *Trifolium repens*. Chem. Nat. Compd..

[16] Joselin J., Brintha T.S.S., Florence A.R., Jeeva S. (2012). Screening of select ornamental flowers of the family *Apocynaceae* for phytochemical constituents. Asian Pac. J. Trop. Dis..

[17] Wang S., Tang F., Yue Y., Yao X., Wei Q., Yu J. (2013). Simultaneous Determination of 12 Coumarins in *Bamboo* Leaves by HPLC. J. AOAC Int..

[18] Nguyen P.-H., Zhao B.T., Kim O., Lee J.H., Choi J.S., Min B.S., Woo M.H. (2016). Anti-inflammatory terpenylated coumarins from the leaves of *Zanthoxylum schinifolium* with α-glucosidase inhibitory activity. J. Nat. Med..

[19] Cho J.-Y., Hwang T.-L., Chang T.-H., Lim Y.-P., Sung P.-J., Lee T.-H., Chen J.-J. (2012). New coumarins and anti-inflammatory constituents from *Zanthoxylum avicennae*. Food Chem..

[20] Sakunpak A., Matsunami K., Otsuka H., Panichayupakaranant P. (2013). Isolation of new monoterpene coumarins from *Micromelum minutum* leaves and their cytotoxic activity against *Leishmania major* and cancer cells. Food Chem..

[21] Petruľová-Poracká V., Repcak M., Vilkova M., Imrich J. (2013). Coumarins of *Matricaria chamomilla* L.: Aglycones and glycosides. Food Chem..

[22] Aziz S.S.S.A., Sukari M.A., Rahmani M., Kitajima M., Aimi N., Ahpandi N.J. (2010). Coumarins from *Murraya paniculata* (Rutaceae). Malays. J. Anal. Sci..

[23] Sun J., Yue Y.-D., Tang F., Guo X.-F. (2010). Coumarins from the leaves of *Bambusa pervariabilis* McClure. J. Asian Nat. Prod. Res..

[24] Li Z.-L., Li Y., Qin N.-B., Li D.-H., Liu Z.-G., Liu Q., Hua H.-M. (2016). Four new coumarins from the leaves of *Calophyllum inophyllum*. Phytochem. Lett..

[25] Razavi S.M., Imanzadeh G., Davari M. (2010). Coumarins from *Zosima absinthifolia* seeds, with allelopatic effects. Eur. Asian J. Biosci..

[26] Li G., Li X., Cao L., Zhang L., Shen L., Zhu J., Wang J., Si J. (2015). Sesquiterpene coumarins from seeds of *Ferula sinkiangensis*. Fitoterapia.

[27] Li G., Wang J., Li X., Cao L., Lv N., Chen G., Zhu J., Si J. (2015). Two new sesquiterpene coumarins from the seeds of *Ferula sinkiangensis*. Phytochem. Lett..

[28] Dien P.H., Nhan N.T., Le Thuy H.T., Quang D.N. (2012). Main constituents from the seeds of Vietnamese *Cnidium monnieri* and cytotoxic activity. Nat. Prod. Res..

[29] Dugrand A., Olry A., Duval T., Hehn A., Froelicher Y., Bourgaud F., Dugrand-Judek A. (2013). Coumarin and Furanocoumarin Quantitation in Citrus Peel via Ultraperformance Liquid Chromatography Coupled with Mass Spectrometry (UPLC-MS). J. Agric. Food Chem..

[30] Dhanavade M.J., Jalkute C.B., Ghosh J.S., Sonawane K.D. (2011). Study antimicrobial activity of lemon (*Citrus lemon* L.) peel extract. Br. J. Pharm. Toxicol..

[31] Miyake Y., Hiramitsu M. (2011). Isolation and extraction of antimicrobial substances against oral bacteria from lemon peel. J. Food Sci. Technol..

[32] Sasidharan S., Chen Y., Saravanan D., Sundram K.M., Latha L.Y. (2011). Extraction, isolation and characterization of bioactive compounds from plants’ extracts. Afr. J. Tradit. Complement. Altern. Med..

[33] Vekariya R.H., Patel H.D. (2014). Recent advances in the synthesis of coumarin derivatives via *Knoevenagel condensation*: A review. Synth. Commun..

[34] He X., Yan Z., Hu X., Zuo Y., Jiang C., Jin L., Shang Y. (2014). FeCl 3-Catalyzed Cascade Reaction: An Efficient Approach to Functionalized Coumarin Derivatives. Synth. Commun..

[35] Asif M. (2015). Pharmacologically potentials of different substituted coumarin derivatives. Chem. Int..

[36] Barot K.P., Jain S.V., Kremer L., Singh S., Ghate M.D. (2015). Recent advances and therapeutic journey of coumarins: Current status and perspectives. Med. Chem. Res..

[37] Dighe N.S., Pattan S.R., Dengale S.S., Musmade D.S., Shelar M., Tambe V., Hole M.B. (2010). Synthetic and pharmacological profiles of coumarins: A review. Sch. Res. Libr..

[38] Abdou M.M. (2017). 3-Acetyl-4-hydroxycoumarin: Synthesis, reactions and applications. Arab. J. Chem..

[39] Al-Ayed A.S. (2011). Synthesis, Spectroscopy and Electrochemistry of New 3-(5-Aryl-4,5-Dihydro-1H-Pyrazol-3-yl)-4-Hydroxy-2H-Chromene-2-One 4, 5 as a Novel Class of Potential Antibacterial and Antioxidant Derivatives. Int. J. Org. Chem..

[40] Al-Majedy Y.K., Kadhum A.A.H., Al-Amiery A.A., Mohamad A.B. (2017). Coumarins: The Antimicrobial agents. Syst. Rev. Pharm..

[41] Aslam K.K., Khosa M.K., Jahan N., Nosheen S. (2010). Short communication: Synthesis and applications of Coumarin. Pak. J. Pharm. Sci..

[42] Liu H., Ren Z.-L., Wang W., Gong J.-X., Chu M.-J., Ma Q.-W., Wang J.-C., Lv X.-H. (2018). Novel coumarin-pyrazole carboxamide derivatives as potential topoisomerase II inhibitors: Design, synthesis and antibacterial activity. Eur. J. Med. Chem..

[43] Sahoo J., Kumar P.S., Mekap S.K. (2015). Synthesis, spectral characterization of some new 3-heteroaryl azo 4-hydroxy coumarin derivatives and their antimicrobial evaluation. J. Taibah Univ. Sci..

[44] Vekariya R.H., Patel K.D., Rajani D.P., Rajani S.D., Patel H.D. (2017). A one pot, three component synthesis of coumarin hybrid thiosemicarbazone derivatives and their antimicrobial evolution. J. Assoc. Arab. Univ. Basic Appl. Sci..

[45] Basanagouda M., Shivashankar K., Kulkarni M.V., Rasal V.P., Patel H., Mutha S.S., Mohite A.A. (2010). Synthesis and antimicrobial studies on novel sulfonamides containing 4-azidomethyl coumarin. Eur. J. Med. Chem..

[46] Soni J.N., Soman S.S. (2014). Reactions of coumarin-3-carboxylate, its crystallographic study and antimicrobial activity. Pharma Chem..

[47] Vyas K.B., Nimavat K.S., Jani G.R., Hathi M.V. (2009). Synthesis and antimicrobial activity of coumarin derivatives metal complexes: An in vitro evaluation. Orbital Electron. J. Chem..

[48] Wei Y., Li S.Q., Hao S.H. (2018). New angular oxazole-fused coumarin derivatives: Synthesis and biological activities. Nat. Prod. Res..

[49] Al-Amiery A.A., Al-Majedy Y.K., Kadhum A.A.H., Mohamad A.B. (2015). Novel macromolecules derived from coumarin: Synthesis and antioxidant activity. Sci. Rep..

[50] Matos M.J., Mura F., Vazquez-Rodriguez S., Borges F., Santana L., Uriarte E., Olea-Azar C. (2015). Study of Coumarin-Resveratrol Hybrids as Potent Antioxidant Compounds. Molecules.

[51] Pérez-Cruz K., Moncada-Basualto M., Morales-Valenzuela J., Barriga-González G., Navarrete-Encina P., Núñez-Vergara L., Squella J., Olea-Azar C., Barriga G. (2018). Synthesis and antioxidant study of new polyphenolic hybrid-coumarins. Arab. J. Chem..

[52] Nagamallu R., Srinivasan B., Ningappa M.B., Kariyappa A.K. (2016). Synthesis of novel coumarin appended bis(formylpyrazole) derivatives: Studies on their antimicrobial and antioxidant activities. Bioorg. Med. Chem. Lett..

[53] Salem M.A.I., Marzouk M.I., El-Kazak A.M. (2016). Synthesis and Characterization of Some New Coumarins with in Vitro Antitumor and Antioxidant Activity and High Protective Effects against DNA Damage. Molecules.

[54] Anand P., Singh B., Singh N. (2012). A review on coumarins as acetylcholinesterase inhibitors for Alzheimer’s disease. Bioorg. Med. Chem..

[55] Bagheri S.M., Khoobi M., Nadri H., Moradi A., Emami S., Jalili-Baleh L., Jafarpour F., Moghadam F.H., Foroumadi A., Shafiee A. (2015). Synthesis and Anticholinergic Activity of 4-hydroxycoumarin Derivatives Containing Substituted Benzyl-1,2,3-triazole Moiety. Chem. Boil. Drug Des..

[56] Razavi S.F., Khoobi M., Nadri H., Sakhteman A., Moradi A., Emami S., Foroumadi A., Shafiee A. (2013). Synthesis and evaluation of 4-substituted coumarins as novel acetylcholinesterase inhibitors. Eur. J. Med. Chem..

[57] Chen L.Z., Sun W.W., Bo L., Wang J.Q., Xiu C., Tang W.J., Shi J.B., Zhou H.P., Liu X.H. (2017). New arylpyrazoline-coumarins: Synthesis and anti-inflammatory activity. Eur. J. Med. Chem..

[58] Pu W., Lin Y., Zhang J., Wang F., Wang C., Zhang G. (2014). 3-Arylcoumarins: Synthesis and potent anti-inflammatory activity. Bioorg. Med. Chem. Lett..

[59] Olmedo D., Sancho R., Bedoya L.M., López-Pérez J.L., Del Olmo E., Muñoz E., Alcami J., Gupta M.P., Feliciano A.S. (2012). 3-Phenylcoumarins as Inhibitors of HIV-1 Replication. Molecules.

[60] Emami S., Dadashpour S. (2015). Current developments of coumarin-based anti-cancer agents in medicinal chemistry. Eur. J. Med. Chem..

[61] Keri R.S., Sasidhar B.S., Nagaraja B.M., Santos M.A. (2015). Recent progress in the drug development of coumarin derivatives as potent antituberculosis agents. Eur. J. Med. Chem..

[62] Akoudad S., Darweesh S.K., Leening M.J., Koudstaal P.J., Hofman A., Van Der Lugt A., Stricker B.H., Ikram M.A., Vernooij M.W. (2014). Use of Coumarin Anticoagulants and Cerebral Microbleeds in the General Population. Stroke.

[63] Hassan M.Z., Osman H., Ali M.A., Ahsan M.J., Ahsan M.J. (2016). Therapeutic potential of coumarins as antiviral agents. Eur. J. Med. Chem..

[64] Wijayabandara M.D.J., Choudhary M.I., Adhikari A. (2015). Characterization of an anti-hyperglycemic coumarin from the fruits of Averrhoa. P. Ann. Sci. Sess. Fac. Med. Sci..

[65] Keshavarzipour F., Tavakol H. (2016). The synthesis of coumarin derivatives using choline chloride/zinc chloride as a deep eutectic solvent. J. Iran. Chem. Soc..

[66] Phadtare S.B., Shankarling G.S. (2012). Greener coumarin synthesis by Knoevenagel condensation using biodegradable choline chloride. Environ. Chem. Lett..

[67] Mi X., Wang C., Huang M., Wu Y., Wu Y. (2014). Preparation of 3-Acyl-4-arylcoumarins via Metal-Free Tandem Oxidative Acylation/Cyclization between Alkynoates with Aldehydes. J. Org. Chem..

[68] Suljić S., Pietruszka J. (2014). Synthesis of 3-Arylated 3,4-Dihydrocoumarins: Combining Continuous Flow Hydrogenation with Laccase-Catalysed Oxidation. Adv. Synth. Catal..

[69] Brahmachari G. (2015). Room Temperature One-Pot Green Synthesis of Coumarin-3-carboxylic Acids in Water: A Practical Method for the Large-Scale Synthesis. ACS Sustain. Chem. Eng..

[70] Pinto L.D.S., De Souza M.V.N. (2017). Sonochemistry as a General Procedure for the Synthesis of Coumarins, Including Multigram Synthesis. Synthesis.

[71] Khan D., Mukhtar S., Alsharif M.A., Alahmdi M.I., Ahmed N. (2017). PhI(OAc) 2 mediated an efficient Knoevenagel reaction and their synthetic application for coumarin derivatives. Tetrahedron Lett..

[72] Fiorito S., Taddeo V.A., Genovese S., Epifano F. (2016). A green chemical synthesis of coumarin-3-carboxylic and cinnamic acids using crop-derived products and waste waters as solvents. Tetrahedron Lett..

[73] Ghomi J.S., Akbarzadeh Z. (2018). Ultrasonic accelerated Knoevenagel condensation by magnetically recoverable MgFe2O4 nanocatalyst: A rapid and green synthesis of coumarins under solvent-free conditions. Ultrason. Sonochem..

[74] Sairam M., Saidachary G., Raju B.C. (2015). Condensation of salicylaldehydes with ethyl 4, 4, 4-trichloro-3-oxobutanoate: A facile approach for the synthesis of substituted 2H-chromene-3-carboxylates. Tetrahedron Lett..

[75] Augustine J.K., Bombrun A., Ramappa B., Boodappa C. (2012). An efficient one-pot synthesis of coumarins mediated by propylphosphonic anhydride (T3P) via the Perkin condensation. Tetrahedron Lett..

[76] He X., Shang Y., Zhou Y., Yu Z., Han G., Jin W., Chen J. (2015). Synthesis of coumarin-3-carboxylic esters via FeCl3-catalyzed multicomponent reaction of salicylaldehydes, Meldrum’s acid and alcohols. Tetrahedron.

[77] Jiang S., Gao J., Han L. (2016). One-pot catalyst-free synthesis of 3-heterocyclic coumarins. Res. Chem. Intermediat..

[78] Li X.T., Liu Y.H., Liu X., Zhang Z.H. (2015). Meglumine catalyzed one-pot, three-component combinatorial synthesis of pyrazoles bearing a coumarin unit. RSC Adv..

[79] Ali M.A.E.A.A. (2015). Bismuth triflate: A highly efficient catalyst for the synthesis of bio-active coumarin compounds via one-pot multi-component reaction. Chin. J. Catal..

[80] Murugavel G., Punniyamurthy T. (2015). Microwave-assisted copper-catalyzed four-component tandem synthesis of 3-N-sulfonylamidine coumarins. J. Org. Chem..

[81] Osman H., Arshad A., Lam C.K., Bagley M.C. (2012). Microwave-assisted synthesis and antioxidant properties of hydrazinyl thiazolyl coumarin derivatives. Chem. Central J..

[82] Ghanei-Nasab S., Khoobi M., Hadizadeh F., Marjani A., Moradi A., Nadri H., Emami S., Foroumadi A., Shafiee A. (2016). Synthesis and anticholinesterase activity of coumarin-3-carboxamides bearing tryptamine moiety. Eur. J. Med. Chem..

[83] Rabahi A., Makhloufi-Chebli M., Hamdi S.M., Silva A.M., Kheffache D., Boutemeur-Kheddis B., Hamdi M. (2014). Synthesis and optical properties of coumarins and iminocoumarins: Estimation of ground- and excited-state dipole moments from a solvatochromic shift and theoretical methods. J. Mol. Liq..

[84] Phadtare S.B., Jarag K.J., Shankarling G.S. (2013). Greener protocol for one pot synthesis of coumarin styryl dyes. Dye. Pigment..

[85] Das D.K., Sarkar S., Khan M., Belal M., Khan A.T. (2014). A mild and efficient method for large scale synthesis of 3-aminocoumarins and its further application for the preparation of 4-bromo-3-aminocoumarins. Tetrahedron Lett..

[86] He X., Chen Y.-Y., Shi J.-B., Tang W.-J., Pan Z.-X., Dong Z.-Q., Song B.-A., Li J., Liu X.-H. (2014). New coumarin derivatives: Design, synthesis and use as inhibitors of hMAO. Bioorg. Med. Chem..

[87] Phakhodee W., Duangkamol C., Yamano D., Pattarawarapan M. (2017). Ph3P/I2-Mediated Synthesis of 3-Aryl-Substituted and 3,4-Disubstituted Coumarins. Synlett.

[88] Sripathi S.K., Logeeswari K. (2013). Synthesis of 3-Aryl Coumarin Derivatives Using Ultrasound. Int. J. Org. Chem..

[89] Sashidhara K., Palnati G., Avula S., Kumar A. (2012). Efficient and General Synthesis of 3-Aryl Coumarins Using Cyanuric Chloride. Synlett.

[90] Rahmani-Nezhad S., Khosravani L., Saeedi M., Divsalar K., Firoozpour L., Pourshojaei Y., Sarrafi Y., Nadri H., Moradi A., Mahdavi M. (2015). Synthesis and Evaluation of Coumarin–Resveratrol Hybrids as 15-Lipoxygenaze Inhibitors. Synth. Commun..

[91] Chandrasekhar S., Kumar H.V. (2015). ChemInform Abstract: An Expeditious Coumarin Synthesis via a “Pseudocycloaddition” Between Salicylaldehydes and Ketene. Synth. Commun..

[92] Fiorito S., Genovese S., Taddeo V.A., Epifano F. (2015). Microwave-assisted synthesis of coumarin-3-carboxylic acids under ytterbium triflate catalysis. Tetrahedron Lett..

[93] Kiyani H., Daroonkala M.D. (2015). A cost-effective and green aqueous synthesis of 3-substituted coumarins catalyzed by potassium phthalimide. Bull. Chem. Soc. Ethiop..

[94] Lieu T.N., Nguyen K.D., Le D.T., Truong T., Phan N.T.S. (2016). Application of iron-based metal–organic frameworks in catalysis: Oxidant-promoted formation of coumarins using Fe 3 O(BPDC) 3 as an efficient heterogeneous catalyst. Catal. Sci. Technol..

[95] Sharma D., Makrandi J.K. (2014). Iodine-mediated one-pot synthesis of 3-cyanocoumarins and 3-cyano-4-methylcoumarins. J. Serb. Chem. Soc..

[96] Rezaei R., Farjam M.H., Farasat M. (2014). Coumarin synthesis via Pechmann condensation utilizing starch sulfuric acid as a green and efficient catalyst under solvent-free conditions. Org. Chem. Indian J..

[97] Pornsatitworakul S., Boekfa B., Maihom T., Treesukol P., Namuangruk S., Jarussophon S., Limtrakul J. (2017). The coumarin synthesis: A combined experimental and theoretical study. Mon. Chem. Chem. Mon..

[98] Bouasla S., Amaro-Gahete J., Esquivel D., López M.I., Jiménez-Sanchidrián C., Teguiche M., Romero-Salguero F.J. (2017). Coumarin Derivatives Solvent-Free Synthesis under Microwave Irradiation over Heterogeneous Solid Catalysts. Molecules.

[99] Mirosanloo A., Zareyee D., Khalilzadeh M.A. (2018). Recyclable cellulose nanocrystal supported Palladium nanoparticles as an efficient heterogeneous catalyst for the solvent-free synthesis of coumarin derivatives via von Pechmann condensation. Appl. Organomet. Chem..

[100] Pakdel S., Akhlaghinia B., Mohammadinezhad A. (2019). Fe3O4@Boehmite-NH2-CoII NPs: An Environment Friendly Nanocatalyst for Solvent Free Synthesis of Coumarin Derivatives Through Pechmann Condensation Reaction. Chem. Afr..

[101] Prateeptongkum S., Duangdee N., Thongyoo P. (2015). Facile iron (III) Chloride Hexahydrate Catalyzed Synthesis of Coumarins.

[102] Mokhtary M., Najafizadeh F. (2012). Polyvinylpolypyrrolidone-bound boron trifluoride (PVPP-BF3); a mild and efficient catalyst for synthesis of 4-metyl coumarins via the Pechmann reaction. Comptes Rendus Chim..

[103] Moradi L., Rabiei K., Belali F. (2016). ChemInform Abstract: Meglumine Sulfate Catalyzed Solvent-Free One-Pot Synthesis of Coumarins under Microwave and Thermal Conditions. Synth.Commun..

[104] Amoozadeh A., Ahmadzadeh M., Kolvari E. (2012). Easy access to coumarin derivatives using alumina sulfuric acid as an efficient and reusable catalyst under solvent-free conditions. J. Chem..

[105] Karimi-Jaberi Z., Masoudi B., Rahmani A., Alborzi K. (2017). Triethylammonium Hydrogen Sulfate [Et 3 NH][HSO 4] as an Efficient Ionic Liquid Catalyst for the Synthesis of Coumarin Derivatives. Polycycl. Aromat. Compd..

[106] Hojati S.F., Hadadnia Z. (2016). A New Highly Efficient Approach to the Synthesis of Coumarin and Its Derivatives. Jordan J. Chem..

[107] Prousis K.C., Avlonitis N., Heropoulos G.A., Calogeropoulou T. (2014). FeCl3-catalysed ultrasonic-assisted, solvent-free synthesis of 4-substituted coumarins. A useful complement to the Pechmann reaction. Ultrason. Sonochem..

[108] Nazeruddin G., Pandharpatte M., Mulani K. (2012). PEG-SO3H: A mild and efficient recyclable catalyst for the synthesis of coumarin derivatives. Comptes Rendus Chim..

[109] Elgogary S.R., Hashem N.M., Khodeir M.N. (2015). Synthesis and Photooxygenation of Linear and Angular Furocoumarin Derivatives as a Hydroxyl Radical Source: Psoralen, Pseudopsoralen, Isopseudopsoralen, and Allopsoralen. J. Heterocycl. Chem..

[110] Sun Y.-F., Liu J.-M., Sun J., Huang Y.-T., Lu J., Li M.-M., Jin N., Dai X.-F., Fan B. (2018). One-Pot Synthesis of Coumarins Unsubstituted on the Pyranic Nucleus Catalysed by a Wells–Dawson Heteropolyacid (H6P2W18O62). Preprints.

[111] Zhu F., Wu X.-F. (2018). Selectivity Controlled Palladium-Catalyzed Carbonylative Synthesis of Propiolates and Chromenones from Phenols and Alkynes. Org. Lett..

[112] Li J., Chen H., Zhang-Negrerie D., Du Y., Zhao K. (2013). Synthesis of coumarins via PIDA/I2-mediated oxidative cyclization of substituted phenylacrylic acids. RSC Adv..

[113] Yan K., Yang D., Wei W., Wang F., Shuai Y., Li Q., Wang H. (2015). Silver-Mediated Radical Cyclization of Alkynoates and α-Keto Acids Leading to Coumarins via Cascade Double C–C Bond Formation. J. Org. Chem..

[114] Liu T., Ding Q., Zong Q., Qiu G. (2015). Radical 5-exo cyclization of alkynoates with 2-oxoacetic acids for synthesis of 3-acylcoumarins. Org. Chem. Front..

